# ‘Re‐Wilding’ an Animal Model With Microbiota Shifts Immunity and Stress Gene Expression During Infection

**DOI:** 10.1111/mec.17586

**Published:** 2024-11-12

**Authors:** Ian Will, Emily J. Stevens, Thomas Belcher, Kayla C. King

**Affiliations:** ^1^ Department of Biology University of Oxford Oxford UK; ^2^ School of Life Sciences Keele University Newcastle‐under‐Lyme UK; ^3^ Jenner Institute University of Oxford Oxford UK; ^4^ Department of Zoology University of British Columbia Vancouver Canada; ^5^ Department of Microbiology & Immunology University of British Columbia Vancouver Canada

**Keywords:** emerging disease, gene expression, host–pathogen, microbiota

## Abstract

The frequency of emerging disease is growing with ongoing human activity facilitating new host–pathogen interactions. Novel infection outcomes can also be shaped by the host microbiota. *Caenorhabditis elegans* nematodes experimentally colonised by a wild microbiota community and infected by the widespread animal pathogen, *Staphylococcus aureus*, have been shown to suffer higher mortality than those infected by the pathogen alone. Understanding the host responses to such microbiota–pathogen ecological interactions is key to pinpointing the mechanism underlying severe infection outcomes. We conducted transcriptomic analyses of *C. elegans* colonised by its native microbiota, *S. aureus* and both in combination. Correlations between altered collagen gene expression and heightened mortality in co‐colonised hosts suggest the microbiota modified host resistance to infection. Furthermore, microbiota colonised hosts showed increased expression of immunity genes and variable expression of stress response genes during infection. Changes in host immunity and stress response could encompass both causes and effects of severe infection outcomes. ‘Re‐wilding’ this model nematode host with its native microbiota indicated that typically commensal microbes can mediate molecular changes in the host that are costly when challenged by a novel emerging pathogen.

## 1 Introduction

Emerging novel host–pathogen interactions are predicted to rise (Carlson et al. 2022; Chen et al. 2011; Clark, Drovetski, and Voelker 2020; Xu et al. 2023). Complicating the already challenging task of predicting emerging disease severity (Anderson et al. 2004; Bersacola et al. 2021; Bonneaud and Longdon 2020; Burge et al. 2014; Fey et al. 2015), hosts carry resident microbiota with the potential to alter infection outcomes (Armitage et al. 2022; Drew, Stevens, and King 2021; Stevens, Bates, and King 2021). Microbiota communities can indirectly shape host–pathogen interactions by modifying host development prior to infection (Dirksen et al. 2020; Izhar and Ben‐Ami 2015; Knutie et al. 2017; Laws et al. 2006; Melero, González, and Elena 2023; Vera‐Ponce de León et al. 2021). During infection, hosts often benefit from a competitive microbiota that directly excludes the invading pathogen or from immune‐priming effects that enhance host resistance to infection (Drew, Stevens, and King 2021; Ford and King 2021; Gerardo and Parker 2014; Hoang, Read, and King 2024). Alternatively, the host microbiota could facilitate pathogen invasion or increase host susceptibility, for example, by altering the immunological environment or providing cues and metabolites to invaders (Lustri, Sperandio, and Moreira 2017; Stevens, Bates, and King 2021). Microbe‐mediated immunopathology also appears to be a key characteristic of emerging disease (Bonneaud et al. 2019; Brown, Le Chat, and Taddei 2008; Graham, Allen, and Read 2005; Yang and Yang 2021). Immunopathology arises from excessive immune activation or dysregulated host stress responses (Cheesman et al. 2016; Richardson, Kooistra, and Kim 2010) that upset the balance of costs and benefits of innate immunity (Lazzaro and Tate 2022). Pathogens additionally can disrupt the microbiota, resulting in virulent dysbiosis (Stevens, Bates, and King 2021). Dysbiosis and immunopathology can benefit pathogens, improving their own establishment and transmission in weakened hosts (Best et al. 2012; Graham, Allen, and Read 2005; Long and Boots 2011). Immunopathology can also weaken the microbiota, giving ‘proactive invader’ pathogens a competitive advantage (Brown, Le Chat, and Taddei 2008; Brown, Inglis, and Taddei 2009). Competitively superior pathogens are thus capable of shaping disease ecology and invasion biology dynamics (Bull and Ebert 2008; Ekroth et al. 2021; Marr, Mautz, and Hara 2008; Ross et al. 2010; Rushton et al. 2000; Torchin et al. 2003). The consequences of disease in highly susceptible hosts can be devastating, rapidly killing most of a population (Carella et al. 2019; Fey et al. 2015; Sanderson and Alexander 2020). Host responses to microbiota–pathogen interactions are critical to differentiate disease processes, and ultimately, forecast wildlife health in nature.

We hypothesised that severe infection outcomes during microbiota–pathogen interactions could be mediated by immunopathology and/or altered host susceptibility. In addition to pathogen‐mediated immunopathology, the microbiota can modulate host immunity (Hoang, Read, and King 2024; Hoang and King 2022). Thus, we hypothesised these microbes could also contribute to immunopathology when confronted with a novel pathogen. Here, we explored the molecular underpinnings of host responses to severe infection during microbiota–pathogen co‐colonisation using transcriptomic data. We leveraged a lab animal model (*Caenorhabditis elegans* nematodes) with a representative natural microbiota community (seven bacterial species), and a widespread opportunistic animal pathogen (Bonneaud, Weinert, and Kuijper 2019) (*Staphylococcus aureus* bacteria). The microbiota, taken from the wild‐derived CeMbio *C. elegans‐*associated microbe collection, can enhance host development in *C. elegans* (Dirksen et al. 2020). However, taken together as a community (and three species individually), the microbiota also enhances co‐colonisation virulence (Stevens et al. 2024). ‘Re‐wilding’ this powerful model system with a natural microbiota allowed us to interrogate ecologically relevant immune and stress mechanisms (Flies and Woods 2019; Leung et al. 2018; Oyesola et al. 2023; Oyesola, Souza, and Loke 2022; Zipple, Vogt, and Sheehan 2023). In some cases, these mechanisms could relate to antimicrobial effectors, stress responses or core pathways that regulate immunity (Anderson and Pukkila‐Worley 2020; Martineau, Kirienko, and Pujol 2021). For example, over‐activation of the PMK‐1 immune pathway has a detrimental effect on *C. elegans* and slows their development (Cheesman et al. 2016) and mutants lacking a compensatory anti‐stress mechanism experience high mortality (Richardson, Kooistra, and Kim 2010). As such, we collected RNAseq data from host worms colonised (or not) by the microbiota in the presence (or absence) of pathogen infection. Co‐colonisation immunopathology and increased host susceptibility are not mutually exclusive. We considered increased or decreased expression of different defence genes as possible synergistic, pro‐virulence changes in immunopathology and susceptibility, respectively. Additionally, experimental adaptation of either the microbiota or pathogen within hosts increases the severity of co‐colonisation infection (Stevens et al. 2024). Therefore, we performed a set of evolved‐line RNAseq experiments conducted independently from the ancestral results we primarily report in the main text here. We sought to understand if microbes evolved within *C. elegans* elicited distinct host gene expression patterns that would reveal key mechanisms of severe co‐colonisation infections.

The within‐host environment mediates pathogen–microbiota interactions in several plant (Hacquard et al. 2017; Lebeis et al. 2015; Vannier, Agler, and Hacquard 2019) and animal species (Davoodi and Foley 2020; Lupp et al. 2007; Vonaesch, Anderson, and Sansonetti 2018), including in this *C. elegans* system. In vivo co‐colonisation with the pathogen reduces the abundance of the microbiota community, a pattern not reflected in vitro, outside the host environment (Stevens et al. 2024). *Caenorhabditis elegans* innate immune responses respond to and affect different microbes by varying degrees (Kim and Mylonakis 2012; Madhu et al. 2020; Roeder et al. 2010), which we hypothesised could be the case with co‐colonisation. For example, an abundant class of antimicrobial peptides in *C. elegans*, the saposins, can differ in their response and effect in a bacterial species dependent manner (Madhu et al. 2020; Roeder et al. 2010). Consequently, we sought to understand if altered host immunity correlated to changes in the resident microbiota community. Using host transcriptomes, we identified possible changes in the within‐host environment that could provide the pathogen with a competitive advantage over the microbiota during co‐colonisation.

## 2 Methods

### 2.1 Experimental Organisms

**FIGURE 1:**
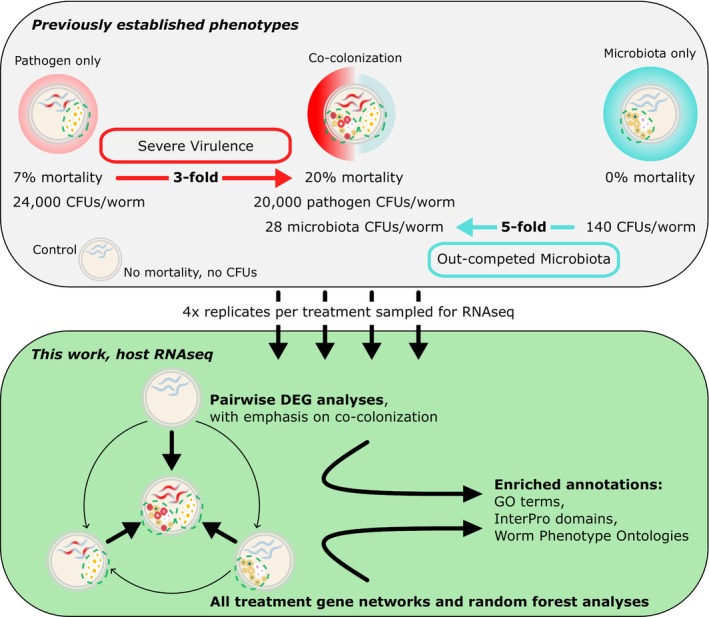
Overview of experimental design and analyses used. Phenotype data are median infection mortality and colony forming units (CFUs) from Stevens et al. (2024). No significant change was reported for pathogen CFUs between pathogen‐only and co‐colonisation treatments, nor was there a difference in mortality between control and microbiota treatments (Stevens et al. 2024). For RNAseq analyses, arrows between treatment types indicate the direction of the DEG analyses. For example, DEGs more highly expressed during co‐colonisation were termed as upregulated relative to control.

The *C. elegans* nematode (germ‐free, isogenic N2 line) was used as a model host, providing an animal host with well‐established genetics and free from any microbes prior to our experimental manipulations. *Escherichia coli* OP50 was used as a food source for *C. elegans*. *Staphylococcus aureus* MSSA476 was used as the bacterial pathogen (Figure 1). The microbiota was assembled from seven bacterial species chosen from the 12‐species CeMbio collection of microbes, which are representative of the most common microbiota components in wild *C. elegans* (Dirksen et al. 2020). Seven species were chosen for practical reasons based on the ability to grow on selective media. These species were: *Pantoea nemavictus* BIGb393, *Lelliottia amnigena* JUb66, *Sphingobacterium paramultivorum* BIGb0170 (synonymous with *Sphingobacterium multivorum* (Wang, Brons, and van Elsas 2021)), *Enterobacter hormaechei* CEent1, *Acinetobacter guillouiae* MYb10, *Pseudomonas lurida* MYb11 and *Ochrobactrum pecoris* MYb71. Experimental evolution of either the microbiota or pathogen was conducted by Stevens et al. (2024) and we detail our use of these strains only in Document S1. Additional details of microbial culturing and worm husbandry can be found in Stevens et al. (2024) and Document S2.

### 2.2 Sampling and RNA Extraction

Experimental infection and colonisation of *C. elegans* was a two‐step process; larvae were reared on *E. coli* or the microbiota, then the young adult worms were transferred to new assay plates with *E. coli*, the microbiota or *S. aureus*. The density of each bacterial species was standardised by optical density (600 nm). The seven microbiota species were pooled in equal amounts to assemble the microbiota community. The rearing plates were saturated with an excess of bacteria (600 μL of microbiota community or *E. coli*), air‐dried and then plated with L1 larval *C. elegans*. Approximately 1000 age‐synchronised L1 larval *C. elegans* worms per plate were fed microbiota or *E. coli* for 48 h, which is sufficient for the microbiota community to colonise the worm gut (Dirksen et al. 2020; Stevens et al. 2024). A minimum of 1000 worms per plate was required to ensure enough RNA could be retrieved for our downstream sequencing methods. We then harvested these young adult worms for infection assays. Worms grown on *E. coli*‐only were transferred to fresh *E. coli* (control) or *S. aureus* (pathogen only) plates. Worms grown on microbiota plates were transferred to either fresh microbiota (microbiota only) or *S. aureus* (co‐colonisation) plates. These assay plates had been inoculated with 100 μL of bacteria and incubated for 24 h to develop a bacterial lawn before the worms were added (microbiota incubated at 20°C, *E. coli* and *S. aureus* at 30°C). Both rearing and assay plates were standard 9 cm Petri dishes. All experimental procedures were conducted in the same laboratory space, with the same organism stocks, by the same researcher as Stevens et al. (2024) to maximise reproducibility and confidently connect our RNAseq data to phenotypic trends described in Stevens et al. (2024).

We incubated four replicates of each assay treatment at 25°C for 12 h to ensure gene expression was captured for live hosts, as substantial mortality was recorded at 24 h in Stevens et al. (2024). Worms were incubated at 25°C as this temperature is efficient for *S. aureus* infection of *C. elegans* and is widely used for such assays (Irazoqui et al. 2010; Sifri et al. 2003). At higher temperatures, adult worms become substantially heat‐stressed (Begasse et al. 2015), but lower temperatures are poor for *S. aureus* growth and virulence. Following assay incubation, we washed all worms off the plates with 3 mL M9 buffer (22 mM KH_2_PO_4_, 22 mM Na_2_HPO_4_, 85 mM NaCl and 1 mM MgSO_4_) with 0.01% Triton‐X and left the worms to settle at the bottom of 15 mL centrifuge tubes. We pipetted 150 μL of the settled worms to 1.5 mL microcentrifuge tubes. We centrifuged the samples for 2 min at 290 × g and then transferred the worms to bead‐bashing tubes in 600 μL RNA lysis buffer (Zymo). Worms were lysed with zircon beads in a Disruptor Genie (2 min, max speed), then centrifuged for 5 min at 16,000 × g. We extracted RNA from the lysate using the Zymo Quick RNA Miniprep kit, as per the manufacturer's protocol. Aliquots (10 μL) were taken from each sample for quality checks and the original samples were immediately frozen at −80°C.

We sent total RNA samples to the University of Liverpool Centre for Genomic Research (CGR) for RNAseq library preparation and Illumina sequencing. The CGR isolated poly‐adenylated mRNA using the NEBNext Poly(A) mRNA Magnetic Isolation Module using 300 ng of input total RNA per sample. In effect, this process selected for eukaryotic host mRNA and carried over only small amounts of microbial RNA. Barcoded libraries were prepared from each sample using the NEBNext Ultra II Directional RNA Library Prep Kit for Illumina with a fragmentation time of 15 min, 12 cycles of amplification, and further purified with Ampure XP beads. Equimolar pools of the libraries were quality checked by qPCR. A 1% PhiX spike‐in was used for sequencing quality control and all libraries were sequenced on an Illumina NovaSeq 6000 on a single lane of a flow cell to produce 150 bp paired‐end reads. Intermediate quality assessment and quantification steps used an Agilent 2100 Bioanalyzer, Agilent 5300 Fragment Analyser, and Qubit. Approximately 15–30 million individual reads were produced per sample. Initial read trimming and quality assessment was also performed by the CGR. Adapter sequences were removed from reads using Cutadapt (v. 1.2.1) with a 3‐bp matching threshold to begin trimming (Martin 2011). The CGR also quality trimmed reads using Sickle (v. 1.200) with Q20 window and minimum read length of 15 bp (Joshi and Fass 2011). These trimmed reads are available on the NCBI Sequence Read Archive, under BioProject PRJNA1100462.

### 2.3 RNAseq Analysis

We pseudo‐mapped and quantified only paired reads to RNA transcript sequences using Salmon (v 1.4.0) with a concatenated multi‐organism transcriptome. Host RNA transcripts were available from NCBI under accession GCF 000002985.6. We generated transcript sequences from genomic annotations for all microbes using GffRead (v. 0.12.7) (Pertea and Pertea 2020) with data from the following NCBI accessions: *S. aureus* (GCF 000013425.1), *E. coli* OP50 (GCF 009496595.1), *A. guillouiae* (GCF 002980175.2), *E. hormaechei* (GCF 014109705.1), *L. amnigena* (GCF 003752235.2), *O. vermis* (GCF 002975205.1), *P. nemavictus* (GCF 013450275.1), *P. lurida* (GCF 002966835.1) and *S. paramultivorum* (GCF 014109745.1). We then excluded all microbial reads from further analysis. Approximately 7–14 million read‐pairs mapped to the host transcriptome remained per sample. We associated remaining transcript names and counts to gene names in the host GFF file (GCF 000002985.6) using tximport (v. 1.30.0) in RStudio (v. 2023.09.1) with R (v. 4.3.2) (R Core Team 2022; RStudio Team 2020; Soneson, Love, and Robinson 2016). Then, we selected only features annotated as mRNA, thereby discarding counts from other transcript sources such as residual rRNA or non‐coding piRNA. We associated genes with functional annotations from *C. elegans* entries from UniProt and StringDB, using a combination of the gene names, UniProt IDs, Wormbase IDs, and StringDB IDs as needed (Bateman et al. 2023; Davis et al. 2022; Szklarczyk et al. 2023).

Using DeSeq2 (v. 1.42.0), we normalised the count data and calculated differential gene expression between experimental treatments (four categories: control, microbiota only, pathogen only and co‐colonisation) (Love, Huber, and Anders 2014). For each of the six possible pairwise contrast of treatments, we selected up‐ and downregulated differentially expressed genes (DEGs) from Wald analyses testing against a null hypothesis of < |1.5|‐fold change in gene expression between treatments (Benjamini–Hochberg adjusted false detection rate of *p* ≤ 0.05). After DeSeq2 independent filtering (missing, low count or outlier data), the number of successfully tested genes per pairwise comparison was in the range of 16,563–17,288. We verified sensible sample clustering by treatment in a principal component analysis (PCA) with DeSeq2 normalised and rlog transformed (option blind = False) gene counts in R.

In a study‐wide comparison of all treatment groups, we selected genes relevant to distinguishing treatments by a modified random forest method tailored to high‐dimensional data using Boruta (v. 8.0.0) in R (Kursa and Rudnicki 2010). Boruta operates by competing variables (genes) against value‐randomised versions of themselves in classification decision trees to determine which genes are informative in distinguishing treatment groups. As Boruta is a heuristic method, we ran three independent iterations and selected genes that were confirmed by at least two iterations (56% of genes selected by any one iteration). Parameters for each iteration were set for our high‐dimensional dataset with 70,000 trees, 900 runs, and the conservative Bonferroni correction for multiple testing (*p* ≤ 0.05 threshold). Prior to running the analyses, we removed all genes that did not have at least one count in at least four samples (17,425 genes used) to limit spurious testing on extremely low count genes.

We created a gene co‐expression network and correlated network modules to each of the experimental treatment types using R package WGCNA (v. 1.72–1) (Langfelder and Horvath 2008). We used the same 17,425 gene set as in the Boruta analysis (above). For WGCNA, we also transformed the expression data with the rlog function of DeSeq2. We constructed a signed‐hybrid gene network using biweight midcorrelation (‘bicor’), with recommended options for binary categorical trait data, a merging cut height of 0.3 and the default minimum module size of 30. After plotting soft power thresholds against scale‐free topology fit and mean connectivity, we selected a soft power threshold of five where topology fit was > 0.9 and mean connectivity < 1000. Hub genes per network module were defined as the top 10 genes with the highest module membership. Significant module eigengene‐treatment correlations were classified by a *p* ≤ 0.05 threshold and we considered ‘strong’ correlations to have a value of at least |0.5|. Module eigengenes explained 68%–23% of gene expression variance (Data S3).

When interpreting host transcriptional responses, we typically assumed significant DEG transcription correlates to translation and functional protein levels (Koussounadis et al. 2015). Alternatively, gene transcription could indicate a homeostatic attempt to counteract fluctuating protein concentrations or, as has been sometimes observed, poorly relate to translation one way or another (Vogel and Marcotte 2012). However, inferring links between gene expression and functional change is necessary to build meaningful mechanistic hypotheses with these data. Furthermore, we pooled nematodes from entire replicate plates to ensure robust RNA extractions, however, this could possibly obscure fine‐scale heterogeneity in bacteria encounter rates and host responses.

### 2.4 Selection of Experiment‐Wide ‘Top Candidate’ Genes and Multi‐Analysis Corroboration of Findings

We used three analytical approaches (see above) to select *C. elegans* genes of interest contributing to increased host mortality and reduced microbiota abundance phenotypes during co‐colonisation. We conducted pairwise differential expression analyses between treatments (DeSeq2), correlated gene co‐expression network modules to treatments (WGCNA) and selected genes with random forests classifying samples by treatment (Boruta). From these three distinct analyses, we were able to consider genes individually, as groups, among all treatments and between specific treatments. Furthermore, when multiple analyses highlighted the same genes, we considered these the most robust candidates representing a true biological signal that may explain the host response underlying phenotypic differences (Stevens et al. 2024).

At least two of the three methods selected many of the same candidate genes underlying host responses to co‐colonisation (Figure S1). Comparing all DEGs from any pairwise treatment comparison including co‐colonisation (*n* = 5354), WGCNA network modules significantly correlated to co‐colonisation (*n* = 7 modules containing 13,727 genes) and all Boruta‐confirmed genes (*n* = 463), produced 5321 (38.4%) genes selected by at least two of the three analyses (Figure S1). Practically, these top candidate genes passing at least two tests were very similar to those selected by differential expression analysis alone. Boruta produced the smallest set of candidate genes, which in part may be due to our best‐of‐three iterations selection criterion and the strict Bonferroni multiple testing adjustment implemented in the Boruta software package. However, Boruta‐selected genes were nearly completely corroborated by the other methods (99.1% of Boruta genes). In contrast, WGCNA grouped genes together by co‐expression patterns, but not every single module member has a strong correlation to treatment type individually, by design. As such, several thousand genes were highlighted by WGCNA alone (8471), but many thousands were also supported as DEGs as well (5278).

### 2.5 Enrichment Analyses to Identify Functional Overrepresentation Within Gene Sets of Interest

For each gene set of interest (e.g., a pairwise DEG subset or a WGCNA network module), we performed a hypergeometric enrichment analysis (multiple testing adjusted FDR *p* ≤ 0.05 and minimum 1.5‐fold increase) to test for overrepresented functional annotations relative to the ‘background’ set of genes from which the set of interest was identified. For DEGs, background genes were the successfully tested genes after DeSeq2 independent filtering for each pairwise comparison. In cases of comparing overlapping DEG results between different treatment pairs, we only used common genes used in all relevant tests. For WGCNA or Boruta, the background pool was the pre‐filtered set of genes with at least one count from at least four samples. Using the stringApp (v. 2.0.2) plugin within Cytoscape (v. 3.10.1) we identified overrepresented Gene Ontology (GO) terms, InterPro protein domains and Worm Phenotype Ontologies (WPO, including direct and inferred evidence) (Doncheva et al. 2019; Paysan‐Lafosse et al. 2023; Schindelman et al. 2011; Shannon et al. 2003; The Gene Ontology Consortium et al. 2023). WPOs terms are limited to some degree by which genes have been interrogated for which functions in worms, and therefore, less comprehensive than the GO and InterPro databases. However, as WPO terms do offer relatively clear, *C. elegans* specific, experimentally supported annotations for host genes, we included analyses of the WPO as one of multiple methods to gain insights to host response in our experiments (Figure 1).

We plotted GO Biological Process and Molecular Function terms in unitless ‘semantic space’ to cluster GO terms by hierarchical relatedness and reduce visual clutter by plotting a representative term for redundant, closely related terms (0.9 similarity threshold) with Revigo (v. 1.8.1) using *C. elegans* GO annotations (Supek et al. 2011). Revigo plot codes were further edited using ggplot2 (v. 3.4.4) in R (Wickham 2016), and labels were selectively applied for readability, informed by the relevance of the term to our discussion, fold‐enrichment and ‘dispensability’ of the term (i.e. a Revigo ranking of the term in how distinct it was from others in the analysis). All figures were finalised with Inkscape (v. 1.3.2).

## 3 Results

### 3.1 Host Immunity, Collagen Biology, Development and Protein Processing Responded to Microbial Colonisation

**FIGURE 2:**
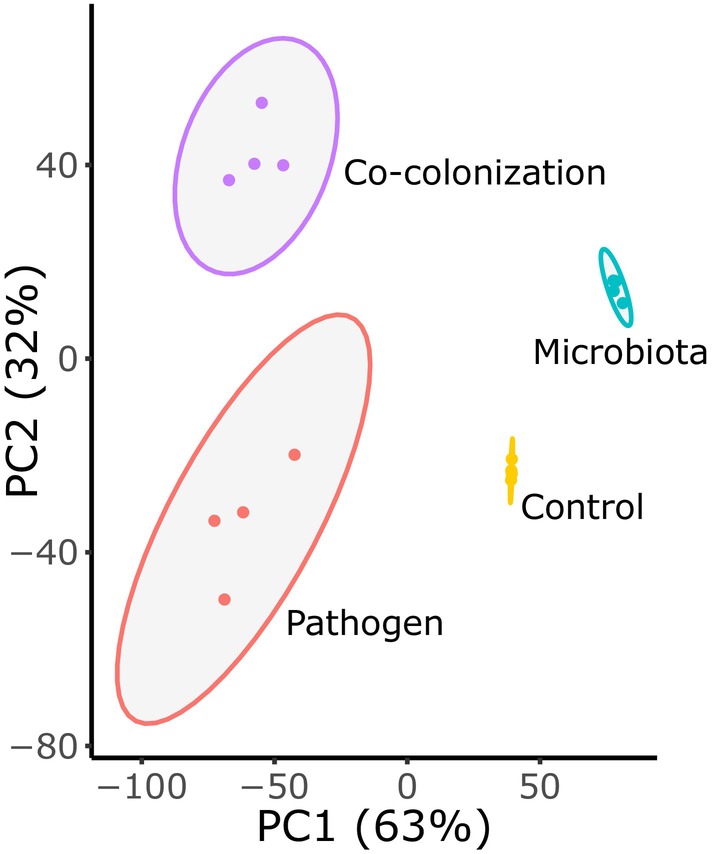
PCA of samples shows a distinct clustering pattern by treatment. PC1 (63% of sample variation) primarily describes the presence or absence of *S. aureus*. PC2 (32%) primarily describes the presence or absence of the microbiota. Shaded ellipses indicate 95% confidence intervals. Each treatment consisted of four samples, colour coded as yellow (control), red (pathogen), blue (microbiome) or purple (co‐colonisation).

**FIGURE 3:**
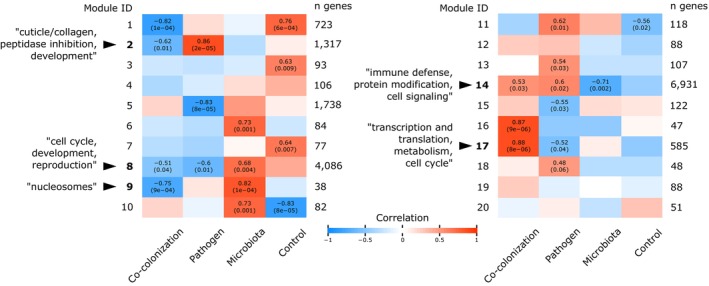
A heatmap of WGCNA network modules shows correlations between modules and treatment type. Seven modules were significantly correlated to co‐colonisation treatment (1, 2, 8, 9, 14, 16, 17) and all member genes were used in the experiment‐wide selection of top DEGs. Of these, modules 2 and 17 were oppositely correlated to pathogen treatment alone, and, 8, 9, and 14 were oppositely correlated to microbiota treatment alone. Co‐colonisation and pathogen treatments did, however, share nearly equal correlations to modules 8 and 14. These modules with contrasting correlations between co‐colonisation and either pathogen or microbiota treatments are labelled in bold and discussed in more detail elsewhere in the text. Significant correlations are annotated on the heatmap with correlation values and associated *p*‐values in parentheses.

**FIGURE 4:**
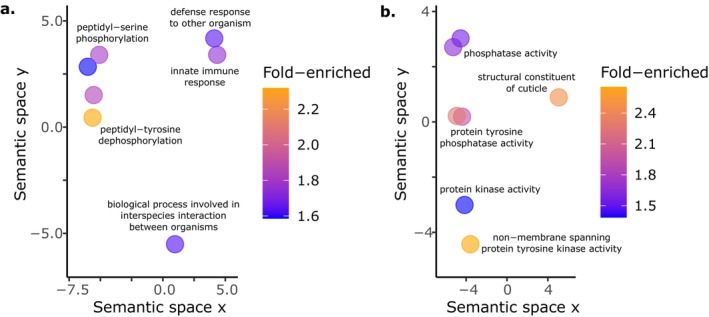
GO terms enriched among top candidate genes selected by at least two analytical methods. Enriched (a) Biological Process and (b) Molecular Function terms reveal changes related to immunity, cuticle/collagen structure and protein modification distinguish the host transcriptional response during *S. aureus* infection, microbiota colonisation and/or co‐colonisation.

**FIGURE 5:**
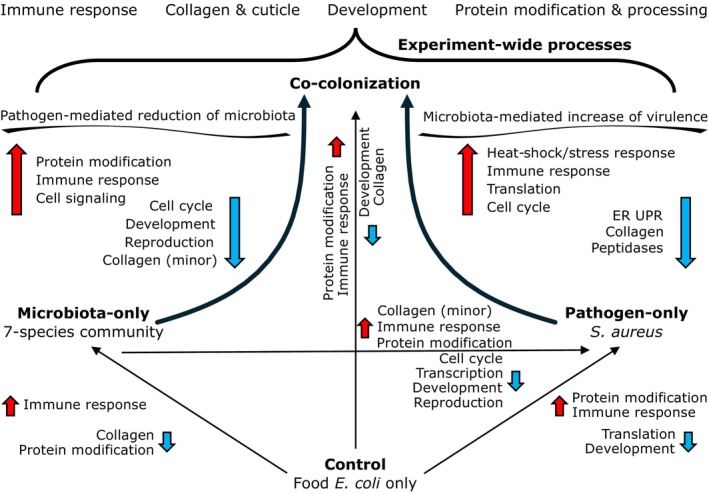
Summary of main results from pairwise comparisons and enriched gene functions. Although genes related to any of the enriched functions could drive enhanced virulence and microbiota suppression, we propose that host immunity, stress response, and collagen biology lend themselves to the clearest hypotheses to explain the observed disease outcomes. Arrow size is for emphasis, not strength of change. Each unique pairwise treatment combination is represented by a single black arrow, indicating the direction of comparison from one treatment to another. Along each black arrow, red and blue arrows indicate upregulated or downregulated processes, respectively. Functional annotations and known gene effects underlie the summary terms we use here. The experiment‐wide processes (top) refer to functions identified using multiple analyses or Boruta.

Host transcriptional profiles were distinct across microbial treatments, revealing candidate processes that shape infection. An unsupervised PCA clearly separated the treatment types (Figure 2). Explaining 63% of the variation in our samples, principal component 1 (PC1) largely distinguished the presence of *S. aureus* infection from pathogen‐free hosts. Also informative, PC2 explained 32% of the variation of our samples, primarily separating hosts colonised by the microbiota or not. The clustering pattern and high amount of variation explained by our treatments suggests that the host genetic response clearly correlates with different infection statuses. Disease outcomes could thus be distinguished, and possibly explained by, host gene expression. Several thousand genes changed expression during co‐colonisation relative to other treatments, identified as DEGs (Table 1), membership in co‐colonisation‐associated gene co‐expression modules (Figure 3), or as important in navigating Boruta decision trees (Figure S1, Data S2). Among top candidate genes supported by at least two analyses (5321 genes) (Figure S1), we detected overrepresentation of immune defence, protein modification and collagen/cuticular structure genes supported by enrichment of GO terms (Figure 4) and InterPro domains (Figure 5, Table S1, Data S2). Similarly, using only the most conservative analysis, Boruta, we found a similar range of functional enrichments related to immunity, collagen and development (Figure 5, Figure S2, Data S2).

**TABLE 1:** Pairwise contrasts and the number of DEGs identified with DeSeq2.

Contrast	Upregulated DEGs	Downregulated DEGs
Control➔Co‐colonisation	2479	1472
Microbiota➔Co‐colonisation	2990	1489
Pathogen➔Co‐colonisation	76	742
Control➔Microbiota	95	1134
Control➔Pathogen	2612	1001
Microbiota➔Pathogen	3720	1523

*Note:* We selected DEGs with a multiple‐testing adjusted FDR threshold of ≤ 0.05 against a null hypothesis of < |1.5|‐fold difference in expression between treatments (Wald test). Arrows indicate direction of change, for example, upregulated DEGs in Control➔Co‐colonisation are more highly expressed during co‐colonisation. We considered co‐colonisation DEGs (first three rows) along with results from the WGCNA and Boruta analyses to produce top candidate genes passing two tests.

### 3.2 Host Stress and Immune Responses Generally Increased During Microbiota and Pathogen Co‐Colonisation

We hypothesised that pathogen infection triggered an extreme host immune response in microbiota‐colonised worms to produce severe virulence. We considered this response as a distinct (but possibly complementary) effect from microbiota‐mediated increased host susceptibility or reduced tolerance (see below). Infection could induce this extreme response directly or indirectly via a pathogen‐dysregulated microbiota (Stevens, Bates, and King 2021). We predicted we would observe hallmark regulatory immune genes and cellular damage genes as DEGs between co‐colonisation and pathogen‐only treatments. We compiled a survey list of experimentally supported and/or well‐reviewed genes (*n* = 63) implicated in immune signalling and regulation, damage response, and reactive oxygen species production (Table S2). In some cases, these genes can have pleiotropic effects on longevity and non‐immune stress responses (Ermolaeva and Schumacher 2014; Mallo et al. 2002; Martineau, Kirienko, and Pujol 2021; Mertenskötter et al. 2013; Rajan et al. 2019; Shivers, Youngman, and Kim 2008; Zheng et al. 2021). These genes included those encoding signal proteins associated with the PMK‐1 (p38 MAPK), MPK‐1, DAF‐2/DAF‐16 (insulin‐like/IGF‐1 signalling), DBL‐1 (TGFβ), KGB‐1 (JNK) and HIF‐1 signalling that we predicted would drive severe and widespread change in the immune response.

Widespread hyperactivation of core immunity pathways did not appear to underpin severe virulence. However, we did find two co‐colonisation DEGs in our shortlist of immunity genes. Both of these DEGs were downregulated compared to pathogen‐only treatment: a transcription factor that responds to pathogen interference with host translation, *zip‐2* (2.4‐fold decrease, W = −3.44, *p* = 0.01) (Martineau, Kirienko, and Pujol 2021; Tran and Luallen 2024) and *sodh‐1* (2.2‐fold decrease, W = −3.3, *p* = 0.02) (Table S2). Gene *sodh‐1*, which supports immunity via reactive oxygen species production, is known to be downregulated in worms colonised with a microbe protective against *S. aureus* (Ford and King 2021; Zhuang et al. 2023).

In contrast to minor reductions of core immune gene expression, severe virulence was tied to strongly and widely upregulated transcription of putative antimicrobials and stress mitigation genes. Among the top 25 upregulated DEGs (of 76), ranked by fold‐increase from pathogen‐only to co‐colonisation, nearly half have roles in or correlate to infection (*clec‐232*, *cld‐1*, F08H9.4, *irg‐4*, *cnc‐6* and *ftn‐1*), stress (*numr‐1*, *numr‐2*, ZC21.10 and *hsp‐12.6*), or otherwise appear to be at least partially regulated by key stress/immune response signalling pathways (*dct‐8* and *dod‐21*) (Figure 5, Table S3). Four of these DEGs were also differentially expressed in an experiment exploring a protective co‐infection against *S. aureus* with *Enterococcus faecalis* (Ford, Drew, and King 2022). Two were similarly upregulated during protective *E. faecalis* co‐infection (*col‐74* and *irg‐4*) and two were downregulated, the opposite of what we observe here (*clec‐232* and *hsp‐12.6*) (Table S3). Furthermore, co‐colonised hosts disproportionally upregulated genes that can alleviate physiological stress and enhance immunity. We performed an enrichment analysis on all 76 co‐colonisation upregulated DEGs to find over‐represented functional annotations. Among this small set of genes, we detected no enriched GO terms, however we did find overrepresented InterPro domains (Table 2, Table S1). Three of these domains were related to small heat shock proteins, annotated to four genes (Figure 5, Table 2). Of these genes *hsp‐12.6* and F08H9.4 were in the top 25 upregulated set (Table S3) and *hsp‐12.3* and *hsp‐16.11* were still clearly expressed three‐fold higher during co‐colonisation compared to pathogen infection alone (w = 4.00 and 3.43, *p* = 0.002 and 0.02, respectively and *hsp‐16.11* also confirmed by Boruta). The other enriched protein domain from this gene set was a ‘CUB‐like domain’. CUB domains are associated with a range of functions, notably with immune responses (Ermolaeva and Schumacher 2014; Shivers, Youngman, and Kim 2008); albeit CUB‐like domains are not as well described. All five genes contributing to this CUB‐like enrichment have been shown to correlate to infection (*cld‐1*, *irg‐4* and F55G11.4) or an immune signalling pathway (*dod‐21* and *dod‐24*) (Fanelli et al. 2023; Madhu, Lakdawala, and Gumienny 2023; Mallo et al. 2002) (Figure 5).

**TABLE 2:** Enriched functional annotations of upregulated DEGs from pathogen infection to co‐colonisation.

Term name	Fold‐enriched	*p*
Alpha crystallin/small heat shock protein, animal type	56	0.0063
Alpha crystallin/Hsp20 domain	56	0.0063
HSP20‐like chaperone	41	0.0063
CUB‐like domain	25	0.0063

*Note:* All enriched terms for this comparison were InterPro domains. Column ‘*p*‐value’ is a multiple‐testing adjusted FDR.

Possibly facilitating protein production and cellular changes in response to severe virulence, co‐colonised hosts activated a gene network module controlling core cellular processes. Module 17 (585 genes) was strongly correlated to co‐colonisation but negatively correlated to pathogen treatment alone (Figure 3). Enrichment analyses returned overrepresentation of numerous cellular functions such as RNA and ribosomal processing, cell cycle, and metabolism (Figure 5, Figure S3, Table S1). Similarly, selecting the top 10 gene module ‘hub genes’ indicated functions related to ribosomes, development and biosynthetic conversion (Data S3). Correspondingly, phenotypes from WPO enrichments indicated fundamental developmental, growth and reproductive effects (Table S1, Data S2).

Microbiota‐colonised hosts appeared to invest in immunity prior to *S. aureus* infection. We found only immune functions enriched among DEGs upregulated by microbiota‐colonised hosts (compared to control worms) (Figure 5, Table S4). Sixteen of these upregulated genes continued to be highly expressed following co‐colonisation, above expression levels observed with the pathogen only (Figure S4a, Data S1). Although a small group of genes, these 16 DEGs included 10 of the top 25 most upregulated co‐colonisation DEGs compared to *S. aureus* infection alone (*cld‐1*, *clec‐232*, *col‐74*, *dod‐21*, *ftn‐1*, *irg‐4*, *nspg‐7.2*, *scl‐25*, *sru‐22* and Y46H3A.5) (Figure S4a, Table S3). This gene expression pattern indicates that the microbiota constitutively induces some of the most dramatically expressed co‐colonised host DEGs, majorly shaping the host response and severe infection outcomes.

### 3.3 Decreased Expression of Host Collagen and ER Unfolded Protein Response Genes Possibly Increased Host Susceptibility to Infection

**FIGURE 6:**
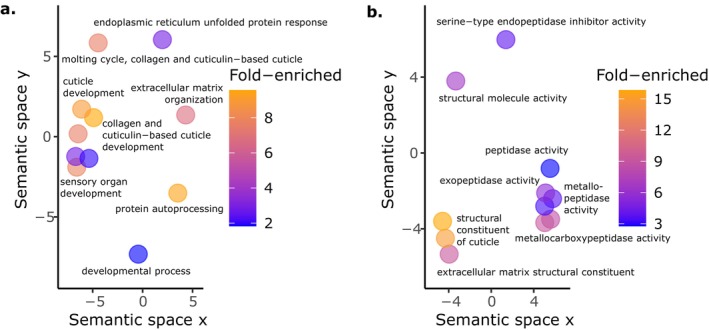
GO term enrichments in DEGs downregulated during co‐colonisation relative to pathogen infection include collagen and cuticle annotations and the ER UPR. Many enriched annotations indicate genes encoding proteins involved with cuticle and collagen development in both (a) Biological Process and (b) Molecular Function are less expressed during co‐colonisation compared to *S. aureus* infection alone. (a) Additionally, the ER UPR is overrepresented among these downregulated DEGs. (b) We also observed reduced expression of peptidase genes (also supported by InterPro domains, Data S2).

Co‐colonised worms strongly downregulated many collagen genes, which may contribute to microbiota‐mediated increases of host susceptibility. Among the top 25 downregulated DEGs (of 742) ranked by fold‐decrease from pathogen‐only to co‐colonisation we found the majority to be cuticle and collagen proteins (Figures 3 and 5, Table S5). The fold‐change of these 25 DEGs ranged from a 899 to 203‐fold decrease, much greater than the range of the top 25 upregulated DEGs from pathogen to co‐colonisation (138 to 5‐fold increase) (Tables S3 and S5). Interestingly, the top 25 downregulated DEGs showed a dramatically different pattern from a protective co‐infection with *E. faecalis* against *S. aureus*, where 17 of these 25 DEGs were instead upregulated (Ford, Drew, and King 2022). Collagen genes were not only strongly, but also broadly, downregulated in our co‐colonised hosts. An enrichment analysis of all 742 downregulated DEGs (from pathogen‐only to co‐colonisation) identified many overrepresented GO terms and InterPro domains related to cuticular biology (Figures 5 and 6, Table S1, Data S2). In addition to supporting a change in cuticular proteins, GO Molecular Functions and InterPro domains also highlighted reduced expression of various peptidases (Figures 5 and 6, Data S2). Our enrichment analysis selected numerous WPO terms—many of which indicate cuticular/locomotor defects or susceptibility to bacterial pathogens associated with reduced gene function (Figure 5, Table S1, Data S2).

Downregulated DEG enrichment also suggested reduced co‐colonised host resistance via a disrupted ‘endoplasmic reticulum unfolded protein response’ (ER UPR). The ER UPR plays a critical role in mitigating stress and immunopathology in *C. elegans* challenged by bacterial gut pathogens or their pore‐forming toxins (Bischof et al. 2008; Martineau, Kirienko, and Pujol 2021; Richardson, Kooistra, and Kim 2010) (Figure S4a). For example, among these downregulated ER UPR genes, we found *abu‐1*, which likely mediates scavenging of unfolding proteins and the ER stress response in worm intestines (Urano et al. 2002).

The presence of the microbiota downregulated the expression of a host gene module that appears to be a coordinated response to pathogen invasion. As this module became dramatically suppressed during co‐colonisation, these hosts may have been unable to properly defend themselves against *S. aureus*. Module 2 (1317 genes) was negatively correlated to co‐colonisation and positively correlated to pathogen‐only treatment (Figure 3). Here, we detected enrichments of cuticle and collagen, peptidases, and development annotations (Figure 5, Figure S5, Table S1). Peptidase annotations included both activation and inactivation, suggesting that proteolytic activity was changing, but not necessarily equally so for all peptidase classes. Worm Phenotype Ontology terms indicated module 2 genes control processes related to the cuticle and morphology that can, in turn, affect processes such as locomotion and bacterial adhesion (Table S1, Data S2). Module 2 hub genes (top 10 by module membership) included a mix of genes such as a predicted antimicrobial, protease inhibitor, reducers of oxidative stress, developmental regulator, and other unclearly annotated genes (Data S3). Furthermore, module 2 contained all 25 of the most downregulated co‐colonisation DEGs (Table S5).

Many of these microbiota‐mediated increases of host susceptibility appear to begin with changes in gene expression before co‐colonisation with *S. aureus*. In the absence of *S. aureus*, microbiota‐only treatment reduced expression of host genes with functions related to collagen and protein modification (relative to controls) (Figure 5, Figure S6, Data S2). A 199 gene subset of microbiota‐modulated DEGs were downregulated both during microbiota‐only (relative to controls) and co‐colonisation (relative to pathogen‐only). These 199 DEGs represent host changes that preceded and persisted though pathogen invasion (Figure S4b). Furthermore, these genes included 22 of the top 25 DEGs most downregulated from pathogen treatment to co‐colonisation (Table S5, Data S1). An enrichment analysis of these 199 DEGs again strongly implicated collagen biology and to a lesser degree, a diminished ER UPR (Figure 5, Tables S1 and S6, Data S2). Additionally, we found WPO terms linking defects in host morphology, locomotion, and pathogen resistance to inhibited expression of these collagen DEGs (Figure 5, Data S2). We discounted the possibility that these DEGs are reflective of a moribund host succumbing to infection, as these genes were downregulated without causing notable host mortality prior to infection. This pattern suggested that, if these DEGs are related to enhanced virulence, they may play a causative role before and during infection.

### 3.4 Pathogen Invasion Reduced Apparent Developmental Benefits of Microbiota and May Create an Immunological Environment Hostile Towards Resident Microbes

Gene expression related to development and reproduction of host worms appear to be both enhanced by the microbiota and hindered by the pathogen and co‐colonisation. Co‐colonisation downregulated host developmental genes compared to hosts colonised by the microbiota alone. Early development and reproduction GO Biological Process terms were the most commonly enriched in this comparison (Figure S7). Overrepresented vitellogenin InterPro domains further implicated depressed reproduction (Data S2). In GO Molecular Function enrichments, we found many transcription‐related terms, suggesting this group of DEGs contained transcription/regulatory factors involved in controlling development processes. Enriched DNA‐binding InterPro domains corroborate these protein functions as well (Data S2).

Also indicating a decrease in development, reproduction, and the cell cycle during co‐colonisation, gene network module 8 (4086 genes) was negatively associated with *S. aureus*. Module 8 was oppositely correlated to the microbiota (positive) and pathogen (negative), but most influenced by the presence of the pathogen during co‐colonisation (negative correlation) (Figure 3). This large gene module was enriched for a wide variety of GO Biological Process and WPO terms related to cell cycle, development, and reproduction—including genes that may serve as transcriptional regulators of these functions (i.e., DNA‐binding annotations in GO Molecular Function terms and InterPro domains) (Figure 5, Figure S9, Table S1, Data S2). The top 10 hub genes largely reflected the overall enriched processes, with genes associated with cuticle biology, reproduction and protein regulation. Another module, module 9 (38 genes), was negatively correlated to co‐colonisation and positively correlated to the microbiota (Figure 3). Module 9 was only enriched for the nucleosome cellular compartment (histone genes *his‐19*, *‐21*, *‐51* and *‐53*) (Data S2).

The in vivo reduction of the microbiota community abundance following *S. aureus* co‐colonisation (Stevens et al. 2024) was correlated to upregulation of protein modification (e.g., protein de‐/phosphorylation) and host immunity (Figure 5, Figure S8, Data S2). In contrast to upregulated immune genes induced and presumably well tolerated by the microbiota alone (Table S4), heightened immunity after pathogen invasion may be capable of impacting the resident microbiota. For example, we found 12 antimicrobial saposins DEGs upregulated from the microbiota alone to co‐colonisation. Notably, none of these saposin genes were upregulated from controls to the microbiota, indicating a unique pathogen‐induced response (Data S1). In agreement with these findings, module 14 (6931 genes) positively correlated to co‐colonisation, indicating increased immunity, protein modification and cell signalling (Figure 5, Figure S10, Table S1, Data S2). The top 10 hub genes largely related to protein modification more so than immunity or cell signalling, when descriptive names/annotations were available (Data S3). Notably, all 12 saposin DEGs upregulated during co‐colonisation were contained within module 14. Module 14 was also positively correlated to pathogen‐only and negatively correlated to microbiota colonisation treatments (Figure 3), again demonstrating contrasting host responses to the different microbes. Although, using experimentally evolved microbe strains, we also detected trends that hint at an additional host‐independent capacity for these microbes to interact and shape virulence (Document S1).

## 4 Discussion

Levels of virulence may deviate from expected evolutionary optima in emerging disease (Bull and Ebert 2008) and infections can drive some of the most damaging mass mortality events (Fey et al. 2015). These ecological impacts may be mediated by three‐way interactions of host, pathogen and microbiota that shape the within‐host ecosystem and virulence (Brown, Inglis, and Taddei 2009; Drew, Stevens, and King 2021; Stevens, Bates, and King 2021; Vannier, Agler, and Hacquard 2019; Vonaesch, Anderson, and Sansonetti 2018). Here, to transition a molecularly tractable model system closer to its natural ecology, we conducted a microbiota re‐wilding experiment on the lab workhorse, *C. elegans*. Using these microbiota‐colonised hosts, we sought to understand the molecular underpinnings of highly virulent infection by a novel pathogen. We correlated severe infection outcomes (established by Stevens et al. 2024), to immune, stress response and collagen gene expression in *C. elegans*. The microbiota may thus be both mediating a hyperactive response to pathogen invasion, as well as reducing host resistance prior to infection.

Increased host stress and immune responses during microbial co‐colonisation coincided with severe virulence mediated by *S. aureus*. The upregulation of stress response genes, especially those encoding small heat shock proteins, may be linked to increased physiological distress (Basha, O'Neill, and Vierling 2012). Although canonically termed ‘heat shock proteins’, many of these proteins respond to a variety of stresses such that host thermal responses and immunity are intertwined on a molecular level (Feder and Hofmann 1999; Sørensen, Kristensen, and Loeschcke 2003). Moreover, the complex interactions of heating and infection can produce diverse outcomes at higher levels of biological organisation, at individual, ecological and evolutionary scales (Cohen et al. 2019; Hector, Gehman, and King 2023; Sauer et al. 2020). At the molecular scale, heat‐shock transcription factor 1 (HSF‐1) and its downstream targets increase host survival when challenged by thermal stress, infection and the effects of aging (Hsu, Murphy, and Kenyon 2003; Singh and Aballay 2006). We found that microbial co‐colonisation upregulated an HSP‐16 family gene (F08H9.4), which has HSF‐1‐binding elements in its promoter and is expressed in the intestine of *C. elegans* (the site of *S. aureus* infection) (Shim, Im, and Lee 2003). Notably, reduced expression of F08H9.4 contributes to increased virulence during infection by the intestinal parasite *Pseudomonas aeruginosa* (Singh and Aballay 2006). We also found co‐colonised hosts increased expression of *hsp‐12.3* and − *12.6*, which respond to immune‐associated signalling pathways (DAF‐2/−16 or PMK‐1) and may play a role in mitigating oxidative stress and promoting longevity in *C. elegans* (Mertenskötter et al. 2013; Murphy et al. 2003). Although upregulation of heat‐shock proteins are elements of an appropriate stress response, extreme levels of a mid‐size heat‐shock protein can have negative fitness consequences (Feder and Hofmann 1999; Ware‐Gilmore et al. 2023). Even if presumably less costly to produce than larger heat‐shock proteins, excessive small heat‐shock protein gene expression may enhance virulence during co‐colonisation rather than combat it.

We hypothesised that a hyperactive immune response associated with co‐colonisation could have facilitated the increased mortality observed in Stevens et al. (2024). Much as an excessive heat‐shock response can impose costs (see above), immunity can lead to immunopathology (Cheesman et al. 2016; Cressler, Graham, and Day 2015; Richardson, Kooistra, and Kim 2010). In re‐wilded laboratory mice, exposed to natural conditions and microbial communities, hosts obtain diverse microbiota and altered immune responses that can enhance parasite burdens (Leung et al. 2018). In our model, hosts re‐wilded with microbiota also suffered more severe infection outcomes. However, we did not find differential gene expression in core immune regulators, such as *pmk‐1*, that would most clearly indicate a centrally controlled and dramatic change in the immune response during co‐colonisation (Cheesman et al. 2016; Martineau, Kirienko, and Pujol 2021; Richardson, Kooistra, and Kim 2010). Individual CeMbio microbiota species and *S. aureus* are known to vary in their activation or repression of a *pmk‐1* reporter (Gonzalez and Irazoqui 2024), possibly explaining how the net effect of co‐colonisation did not alter expression of this pathway. Additionally, microbiota species (without the pathogen) exhibit context dependent virulence in immune‐compromised hosts (Gonzalez and Irazoqui 2024). We propose that less central (possibly smaller) immune costs and latent microbiota pathogenicity lead to sizeable increases in virulence when paired with reduced host resistance (e.g., changes in heat‐shock response, ER UPR and collagen biology). In our study, the microbiota clearly mediated changes in host immunity both before infection and after co‐colonisation with *S. aureus*. However, immune effectors do not affect all pathogens equally (Kim and Mylonakis 2012) and the microbiota induced immune response appears maladaptive against *S. aureus*. Speculatively, effective microbiota immune priming against novel *S. aureus* infections might not have evolved because the microbiota and pathogen lack a coevolutionary history together in *C. elegans*.

Hyper‐immunity may also cause an immunologically hostile environment for resident microbiota, which can promote infections by ‘proactive invaders’ (Brown, Le Chat, and Taddei 2008; Brown, Inglis, and Taddei 2009). In line with this hypothesis, we detected increased co‐colonised host immune gene expression, while Stevens et al. (2024) found reduced microbiota abundances with *S. aureus* infection. Possibly indicating asymmetric effects of immunity on the microbes, we detected the broadly activated *spp‐5* (Roeder et al. 2010), the seemingly more specific *spp‐1* (Alper et al. 2007) and 10 other *spp* DEGs activated by co‐colonisation relative to the microbiota alone. These saposin DEGs offer an example of candidate effectors that could disrupt the microbiota. Furthermore, Stevens et al. (2024) found that *S. aureus* did not change in abundance between single infection or co‐colonisation—suggesting that the pathogen is not perturbed by the increased immune response. Pathogen load does not tell all, however, as virulence and transmission are complex traits, affected by the host (including microbiota), pathogen and environment (Bonneaud et al. 2019; Casadevall and Pirofski 1999; Cressler et al. 2016; Day, Graham, and Read 2007; Hector, Gehman, and King 2023).

The microbiota dramatically reduced *C. elegans* collagen gene expression, revealing possible tradeoffs between developmental rate and susceptibility to invasive pathogens. Nematode development can be shaped by microbes (Kim 2013), with the CeMbio microbiota strains used here increasing host developmental rate (Dirksen et al. 2020). As *C. elegans* develop into late adulthood, they may become more susceptible to bacterial infection, whereas developmentally‐younger, long‐lived mutants are more resistant (Garsin et al. 2003; Laws et al. 2006; Youngman, Rogers, and Kim 2011). In turn, expression of some collagen genes correlate to *C. elegans* age, with many decreasing as adult worms become older (Ewald et al. 2014; Palani et al. 2023; Roux et al. 2023). *Caenorhabditis elegans* collagen genes also change expression following infection and, in some cases, have been demonstrated to affect host resistance (Dodd et al. 2018; Ford, Drew, and King 2022; Sahu et al. 2012; Sellegounder et al. 2019; Wong et al. 2007). Notably, in contrast to the widespread downregulation we found, many *C. elegans* collagen genes are upregulated during microbe‐mediated protection against *S. aureus* infection (Ford, Drew, and King 2022). We hypothesise that microbiota enhanced development alters host collagen and thereby increases *S. aureus* susceptibility.

Through the perspective of host gene expression, we identified candidate processes that may reveal the role of the microbiota in driving extreme disease outcomes. We detected shifts in host immune and stress responses that could be both cause (immunopathology) and effect (response to severe infection). We also found microbiota‐mediated effects on host collagen biology that may reflect benefits (faster development) or inflict costs (infection susceptibility) in a context dependent manner when at risk of invasion by a novel pathogen. Microbes influence development and lifespan in a variety of animals (Erkosar et al. 2013; Sison‐Mangus, Mushegian, and Ebert 2015; Vera‐Ponce de León et al. 2021; Zhang et al. 2013), with quicker development offering possible fitness benefits through host condition and reproduction. However, re‐wilding this model animal–pathogen system with its native microbiota suggests that benefits can become costs during novel pathogen invasion. The ecological scenarios (e.g., high or low risk of pathogen transmission) in which changes to developmental rate and immunity confer net benefits or costs remain to be fully elucidated.

Improved mechanistic hypotheses of how microbiota communities shape host responses and outcomes to novel pathogens can support better models of emerging infectious disease. These models can empower predictions and management of emerging diseases, with broad implications, from terrestrial agriculture to wild marine systems (Anderson et al. 2004; Bersacola et al. 2021; Burge et al. 2014). Furthermore, infection is the primary factor in driving mass mortality events, which can be further exacerbated by additional stressors (Carella et al. 2019; Fey et al. 2015; Sanderson and Alexander 2020). Novel pathogen adaptations that facilitate exploitation of new species are one component of understanding emerging disease (Dowling, Hill, and Bonneaud 2020; Stevens et al. 2024), but a thorough understanding of the conditions that shape host resistance and tolerance, such as their microbiota (Drew, Stevens, and King 2021; Hoang and King 2022; Stevens, Bates, and King 2021) is also critical in keeping pace with a rapidly changing world.

## Author Contributions

K.C.K. and E.J.S. conceived the study. K.C.K. E.J.S. and I.W. designed the study. E.J.S. conducted the evolution experiment and E.J.S. and T.B. conducted the RNA extractions. I.W. processed and analysed the sequencing data. I.W. wrote the first manuscript draft, with revisions from K.C.K. and E.J.S.

## Conflicts of Interest

The authors declare no conflicts of interest.

## Supporting information


**Data S1** Excel master data sheet with each gene and all DESeq2 statistics, Boruta results and WGCNA module. Columns are annotated with descriptive notes.

**Data S2** Excel with tabs for every enrichment analysis, containing outputs from the Cytoscape‐StringApp.

**Data S3** Excel of WGCNA outputs for correlation data, variance explained by module eigengenes, module memberships and additional colour‐to‐number module ID matching.

**Figure S1** Three analytical methods highlight expression of many of the same host genes associated with co‐colonisation.
**Figure S2** GO term enrichments in WGCNA module 17, which is positively correlated to co‐colonisation and negatively correlated to pathogen treatment alone.
**Figure S3** GO terms enriched among genes selected by Boruta, the most conservative experiment‐wide analysis.
**Figure S4** Genes that are uniquely induced by the microbiota even before pathogen invasion may include important players in understanding changes in host condition that underlie the difference in mortality between co‐colonisation and pathogen treatments.
**Figure S5** GO term enrichments in WGCNA module 2, which is negatively correlated to co‐colonisation and positively correlated to pathogen treatment alone.
**Figure S6** GO term enrichments from DEGs downregulated from controls to microbiota colonisation.
**Figure S7** GO term enrichments from DEGs downregulated from microbiota colonisation to co‐colonisation.
**Figure S8** GO term enrichments from DEGs upregulated from microbiota colonisation to co‐colonisation.
**Figure S9** GO term enrichments in WGCNA module 8, which is positively correlated to microbiota treatment and negatively correlated to co‐colonisation pathogen‐only infections.
**Figure S10** GO term enrichments in WGCNA module 14, which is negatively correlated to microbiota treatment and positively correlated to co‐colonisation pathogen‐only infections.
**Document S1**. Experiments with evolved bacteria.
**Document S2**. Culturing and infection assay methods.
**Table S1** Overview of counts of overrepresented functional annotations returned from hypergeometric enrichment analyses.
**Table S2** Short list of reviewed *C. elegans* immune genes with a summary of our results per gene.
**Table S3** The top 25 upregulated DEGs ranked by fold‐increase during co‐colonisation compared to pathogen‐only.
**Table S4** Enriched functional annotations of upregulated DEGs from control to microbiota colonisation.
**Table S5** The top 25 downregulated DEGs ranked by fold‐decrease during co‐colonisation compared to pathogen‐only.
**Table S6** Enriched GO terms among the 199 downregulated DEGs correlated to microbiota colonisation.

## Data Availability

RNAseq reads are available on the NCBI Sequence Read Archive, under BioProject PRJNA1100462.
