# Supplementary material for: ‘Re‐Wilding’ an Animal Model With Microbiota Shifts Immunity and Stress Gene Expression During Infection
Source: Mol Ecol. 2024 Nov 12;34(1):e17586. doi: 10.1111/mec.17586 (PMC11665493; doi:10.1111/mec.17586)
Supplement: Supplementary file 4 — Figure S1 Three analytical methods highlight expression of many of the same host genes associated with co‐colonisation. Figure S2 GO term enrichments in WGCNA module 17, which is positively correlated to co‐colonisation and negatively correlated to pathogen treatment alone. Figure S3 GO terms enriched among genes selected by Boruta, the most conservative experiment‐wide analysis. Figure S4 Genes that are uniquely induced by the microbiota even before pathogen invasion may include important players in understanding changes in host condition that underlie the difference in mortality between co‐colonisation and pathogen treatments. Figure S5 GO term enrichments in WGCNA module 2, which is negatively correlated to co‐colonisation and positively correlated to pathogen treatment alone. Figure S6 GO term enrichments from DEGs downregulated from controls to microbiota colonisation. Figure S7 GO term enrichments from DEGs downregulated from microbiota colonisation to co‐colonisation. Figure S8 GO term enrichments from DEGs upregulated from microbiota colonisation to co‐colonisation. Figure S9 GO term enrichments in WGCNA module 8, which is positively correlated to microbiota treatment and negatively correlated to co‐colonisation pathogen‐only infections. Figure S10 GO term enrichments in WGCNA module 14, which is negatively correlated to microbiota treatment and positively correlated to co‐colonisation pathogen‐only infections. Document S1. Experiments with evolved bacteria. Document S2. Culturing and infection assay methods. Table S1 Overview of counts of overrepresented functional annotations returned from hypergeometric enrichment analyses. Table S2 Short list of reviewed C. elegans immune genes with a summary of our results per gene. Table S3 The top 25 upregulated DEGs ranked by fold‐increase during co‐colonisation compared to pathogen‐only. Table S4 Enriched functional annotations of upregulated DEGs from control to microbiota colonisation. Table S5 The top 25 downregu [file MEC-34-e17586-s002.pdf]

"Re-wilding" an animal model with microbiota shifts immunity and stress gene expression during infection.

Ian Will[1*$], Emily J. Stevens[1,2*], Thomas Belcher[3] and Kayla C. King[1,4,5]

[1] Department of Biology, University of Oxford, Oxford, United Kingdom

[2] School of Life Sciences, Keele University, Newcastle-under-Lyme, United Kingdom

[3] Jenner Institute, University of Oxford, Oxford, United Kingdom

[4] Department of Zoology, University of British Columbia, Vancouver, Canada

[5] Department of Microbiology & Immunology, University of British Columbia, Vancouver, Canada

* equal author contribution

$ corresponding author: ian.will@biology.ox.ac.uk

Running title: Wild microbiota shifts model host gene expression.

Supplemental Information

Supplemental Data separately attached:

Supplemental Data S1. Excel master data sheet with each gene and all DESeq2 statistics, Boruta results, and WGCNA module. Columns are annotated with descriptive notes.

Supplemental Data S2. Excel with tabs for every enrichment analysis, containing outputs from Cytoscape-StringApp.

Supplemental Data S3. Excel of WGCNA outputs for correlation data, variance explained by module eigengenes, module memberships, and additional color-to-number module ID matching.

Contents:

Table S1. Overview of counts of overrepresented functional annotations returned from hypergeometric enrichment analyses.

Table S2. Short list of reviewed *C. elegans* immune genes with a summary of our results per gene.

Table S3. The top 25 upregulated DEGs ranked by fold-increase during co-colonization compared to pathogen-only.

Table S4. Enriched functional annotations of upregulated DEGs from control to microbiota colonization.

Table S5. The top 25 downregulated DEGs ranked by fold-decrease during co-colonization compared to pathogen-only.

Table S6. Enriched GO terms among the 199 downregulated DEGs correlated to microbiota colonization.

Figure S1. Three analytical methods highlight expression of many of the same host genes associated with co-colonization.

Figure S2. GO term enrichments in WGCNA network 17, which is positively correlated to co-colonization and negatively correlated to pathogen treatment alone.

Figure S3. GO terms enriched among genes selected by Boruta, the most conservative experiment-wide analysis.

Figure S4. Genes that are uniquely induced by the microbiota even before pathogen invasion may include important players in understanding changes in host condition that underly the difference in mortality between co-colonization and pathogen treatments.

Figure S5. GO term enrichments in WGCNA network 2, which is negatively correlated to co-colonization and positively correlated to pathogen treatment alone.

Figure S6. GO term enrichments from DEGs downregulated from controls to microbiota colonization.

Figure S7. GO term enrichments from DEGs downregulated from microbiota colonization to co-colonization.

Figure S8. GO term enrichments from DEGs upregulated from microbiota colonization to co-colonization.

Figure S9. GO term enrichments in WGCNA network 8, which is positively correlated to microbiota treatment and negatively correlated to co-colonization pathogen-only infections.

Figure S10. GO term enrichments in WGCNA network 14, which is negatively correlated to microbiota treatment and positively correlated to co-colonization pathogen-only infections.

Supplemental Document S1. Experiments with evolved bacteria.

Supplemental Document S2. Culturing and infection assay methods.

Table S1. Overview of counts of overrepresented functional annotations returned from hypergeometric enrichment analyses. Gene sets are listed in order of results reporting. Abbreviations: n genes (number of genes in set), GO BP (GO Biological Process), GO MF (GO Molecular Function), GO CC (GO Cellular Component), InterPro (InterPro domain), WPO (Worm Phenotype Ontology). These results are all included in the full Supplemental Data S2 data and summarized in the listed "Result summary" locations.

Gene Set	n genes	GO BP	GO MF	GO CC	InterPro	WPO	Result summary
"Top candidates" passing two tests	5,321	9	7	2	13	0	Fig. 4
Boruta confirmed	463	13	6	3	14	0	Fig. S3
Pathogen→Co-colonization, upregulated DEGs	76	0	0	0	4	0	Table 2
Control→Microbiota, upregulated DEGs	95	5	0	0	1	0	Table S4
Pathogen→Co-colonization, downregulated DEGs	742	12	11	10	38	43	Fig. 6
Control→Microbiota, downregulated DEGs	1,134	12	16	4	28	1	Fig. S6
Pathogen→Co-colonization, downregulated DEGs also down from Control→Microbiota	199	7	4	4	2	23	Table S6
Network 17 (positive correlation to co-colonization)	585	80	30	52	5	101	Fig. S2
Network 2 (negative correlation to co-colonization)	1,317	16	11	11	23	36	Fig. S5
Microbiota→Co-colonization, downregulated DEGs	1,489	31	14	11	20	31	Fig. S7
Microbiota→Co-colonization, upregulated DEGs	2,990	15	13	2	22	0	Fig. S8
Network 8 (negative correlation to co-colonization)	4,086	417	29	86	16	461	Fig. S9
Network 14 (positive correlation to co-colonization)	6,931	13	6	4	7	0	Fig. S10

Table S2. Short list of reviewed *C. elegans* immune genes with a summary of our results per gene. Two of these genes are downregulated during co-colonization relative to pathogen infection alone (red shading). With two downregulated and six upregulated during co-colonization relative to control (yellow shading). Genes are ordered by WGCNA network membership.

Gene	WGCNA	Boruta	Control→Co-colonization	Pathogen→Co-colonization
atf-4	1	confirmed	Down	No
cwn-2	1	NA	No	No
sod-1	1	NA	No	No
bli-3	2	NA	No	No
nhr-57	2	NA	No	No
sod-2	3	NA	No	No
cebp-2	5	NA	No	No
hsp-4	5	NA	No	No
zip-4	5	NA	No	No
atf-6	8	NA	No	No
atf-7	8	NA	No	No
ced-3	8	NA	No	No
daf-2	8	NA	No	No
egl-30	8	NA	No	No
hsf-1	8	NA	No	No
hsp-3	8	NA	Down	No
let-60	8	NA	No	No
mek-2	8	NA	No	No
mig-1	8	NA	No	No
mpk-1	8	NA	No	No
pink-1	8	NA	No	No
pmk-1	8	NA	No	No
rab-11.1	8	NA	No	No
rab-5	8	NA	No	No
skn-1	8	NA	No	No
skpo-1	8	NA	No	No
bar-1	14	NA	No	No
cebp-1	14	NA	No	No
daf-16	14	NA	No	No
daf-7	14	confirmed	No	No
dbl-1	14	NA	No	No
dcar-1	14	NA	Up	No
elt-2	14	NA	No	No
flp-18	14	NA	Up	No
flp-21	14	NA	Up	No
fshr-1	14	NA	No	No

gar-2	14	NA	No	No
gar-3	14	NA	No	No
glb-12	14	NA	No	No
hif-1	14	NA	No	No
hlh-30	14	NA	No	No
ins-11	14	NA	Up	No
ins-6	14	NA	No	No
kgb-1	14	NA	No	No
nhr-14	14	NA	No	No
nhr-45	14	NA	No	No
octr-1	14	NA	No	No
pcdr-1	14	NA	No	No
sek-1	14	NA	No	No
sma-2	14	NA	No	No
sma-3	14	NA	No	No
sma-4	14	NA	No	No
sod-4	14	NA	Up	No
sodh-1	14	confirmed	Up	Down
sta-2	14	NA	Up	No
tir-1	14	NA	No	No
zip-2	14	NA	No	Down
nhr-86	17	NA	No	No
pek-1	17	NA	No	No
sphk-1	17	NA	No	No
rab-11.2	19	NA	No	No
jkk-1	20	NA	No	No
sod-3	20	NA	No	No

Table S3. The top 25 upregulated DEGs ranked by fold-increase during co-colonization compared to pathogen-only. Many of these DEGs have reported correlations to or functions in immune and stress response. Eight of the 25 are also supported by Boruta, 18 of the 25 are in WGCNA modules significantly correlated to co-colonization. Two DEGs have shared expression patterns with Ford et al., 2022 during a protective co-infection against *S. aureus* (Up), two have the opposite response (Down), and the rest were not DEGs in that study (N). Network ID numbers are given under column WGCNA and support from the Boruta analysis are given as either yes (Y) or no (N). Fold-changes (FC) are increases from pathogen-only to co-colonization, the p-value is an FDR adjusted p-value, and W indicates the Wald test statistic.

Notes	Gene	WGCNA	Boruta	FC	W	p-value	DEG during protection
Nematode Specific Peptide family 7.2	nspg-7.2	5	N	138	12.50	1.01E-32	N

A Coiled Coil protein	C05D12.4	5	N	103	3.80	3.97E-3	N
DAF-16/FOXO Controlled, germline Tumor affecting 8	*dct-8*	-	N	93	4.83	5.49E-5	N
Collagen 74 – Cuticular protein	*col-74*	10	N	66	3.06	4.66E-2	Up
A FMRF-like Peptide	F59C6.18	17	N	50	3.63	7.62E-3	N
Downstream of DAF-16 21	*dod-21*	17	Y	36	12.44	1.99E-32	N
Serpentine Receptor, class U 22 – Chemosensory GPCR	*sru-22*	17	Y	30	3.85	3.33E-3	N
C-type Lectin 232 – Downregulated during protective co-colonization (Ford et al., 2022)	*clec-232*	5	N	12	5.95	1.59E-7	Down
CUB-like domain 1 – Putatively secreted and upregulated in response to bacterial infection (Fanelli et al., 2023)	*cld-1*	8	Y	11	3.87	3.15E-3	N
A SHSP domain-containing protein – A hsp-16, small heat shock protein primarily expressed in the intestines and some neurons that can support antimicrobial immunity (Shim et al., 2003; Singh & Aballay, 2006)	F08H9.4	14	N	10	3.09	4.29E-2	N
A Cys-rich protein	C54F6.6	14	N	10	3.07	4.45E-2	N
Serpentine Receptor, class I 39 – GPCR	*sri-39*	14	N	8	3.38	1.79E-2	N
Nuclear Localized Metal Responsive 1 – Heavy metal resistance, not shown to respond to heat stress or infection, although heavy metal tolerance does share signaling proteins with bacterial resistance (*e.g.*, via PMK-1, DAF-2, DAF-16) (Barsyte et al., 2001; Kim et al., 2004; Tvermoes et al., 2010)	*numr-1*	17	N	7	6.03	1.02E-7	N
Nuclear Localized Metal Responsive 2 – as *numr-1* (above)	*numr-2*	17	N	7	6.03	1.02E-7	N
A Fungal Lipase-like domain-containing protein – some lipases may play roles in antimicrobial immunity (Roberts et al., 2010; Wong et al., 2007)	Y46H3A.5	17	Y	7	4.87	4.52E-5	N
A Rieske domain-containing protein – Heat shock inducible and HSF-1 regulated, but with unclear function (Brunquell et al., 2016)	ZC21.10	17	N	7	3.12	3.92E-2	N
A DUF19 domain-containing protein	F40G12.5	14	Y	6	5.96	1.44E-7	N
Infection Response Gene 4 - Expressed in intestines and head neurons, downstream of PMK-1, DBL-1, and insulin-like signaling, with a role in bacterial defense (Madhu et al., 2023; Peterson et al., 2019; Pukkila-Worley et al., 2014; Shapira et al., 2006)	*irg-4*	10	Y	6	7.78	8.47E-13	Up
Caenacin (*Caenorhabditis* bacteriocin) 6 – responds to fungi and bacteria (Dierking et al., 2016)	*cnc-6*	14	N	6	3.05	4.77E-2	N
SCP-Like extracellular protein 25	*scl-25*	5	N	6	3.64	7.22E-3	N

Heat Shock Protein 12.6 – A small heat shock protein that is induced by DAF-16 (possibly influenced by PMK-1 as well) and associated with increased longevity, possibly by reducing oxidative stress (Mertenskötter et al., 2013; Murphy et al., 2003)	*hsp-12.6*	14	Y	6	4.36	4.39E-4	Down
Ferritin 1 – A intestinally expressed iron-storage protein that is responsive to PMK-1/ELT-2 and insulin-like (DAF) signaling, *ftn-1* is upregulated in some bacterial infections (Rajan et al., 2019; Romney et al., 2008)	*ftn-1*	14	Y	6	4.29	5.99E-4	N
A CX domain-containing protein	F21C10.19	14	N	5	4.22	7.85E-4	N
A Fibrinogen C-terminal domain-containing protein	F02H6.6	14	N	5	3.08	4.42E-2	N
A Fungus-Induced Protein related protein	F54D8.10	16	N	5	3.75	4.86E-3	N

Table S4. Enriched functional annotations of upregulated DEGs from control to microbiota colonization. Column "p-value" is a multiple-testing adjusted FDR.

Annotation type	Term name	Fold-enriched	p-value
GO Biological Process	Defense response to Gram-positive bacterium	12	2.61E-2
GO Biological Process	Defense response to bacterium	12	2.58E-6
GO Biological Process	Innate immune response	11	8.72E-11
GO Biological Process	Defense response to Gram-negative bacterium	10	1.96E-2
GO Biological Process	Defense response to other organism	9	8.72E-11
InterPro Domains	CUB-like domain	50	2.85E-14

Table S5. The top 25 downregulated DEGs ranked by fold-decrease during co-colonization compared to pathogen-only. Most of these DEGs are collagen and cuticle genes, which can have varying impacts – including susceptibility to infection. Ten of the 25 DEGs are also supported by Boruta, and all are in WGCNA network 2, which is negatively correlated to co-colonization treatment. During protective co-colonization with *E. faecalis*, 17 of these 25 *C. elegans* DEGs are conversely upregulated (Up) and the rest were not DEGs in that study (N) (Ford et al., 2022). Network ID numbers are given under column WGCNA and support from the Boruta analysis are given as either yes (Y) or no (N). Fold-changes (FC) are increases from pathogen-only to co-colonization, p-value is an FDR adjusted p-value, and W indicates the Wald test statistic.

Notes	Gene	WGCNA	Boruta	FC	W	p-value	DEG during protection
Groundhog 6 – hedgehog-like family	grd-6	2	Y	899	-8.45	4.13E-15	N

Collagen 88 – may be modestly induced by heat stress and contribute to longevity (Garrigues et al., 2019; Palani et al., 2023)	col-88	2	Y	716	-8.42	5.60E-15	Up
Blistered cuticle 1 – collagen	bli-1	2	N	539	-11.12	5.11E-26	N
Collagen 161 – downregulated in long-lived, thermally tolerant *npr-8* mutants (Palani et al., 2023)	col-161	2	N	522	-9.76	4.68E-20	Up
Collagen 73 – downregulated in long-lived, thermally tolerant *npr-8* mutants (Palani et al., 2023)	col-73	2	Y	510	-6.34	1.65E-08	Up
A glycine Rich Secreted Protein	Y47D7A.15	2	N	485	-9.87	1.59E-20	N
Squat 1 – collagen	sqt-1	2	N	462	-10.04	3.11E-21	Up
Dumpy 5 – collagen	dpy-5	2	N	395	-9.57	2.73E-19	Up
Collagen 17	col-17	2	Y	361	-11.29	8.98E-27	Up
Collagen 156	col-156	2	Y	358	-7.11	1.02E-10	Up
A DUF281 domain-containing protein	T06G6.6	2	N	351	-7.00	2.29E-10	No
A methyltransferase FkbM domain-containing protein	ZK1025.3	2	Y	344	-7.07	1.40E-10	Up
Dumpy 4 – collagen	dpy-4	2	N	335	-11.44	1.60E-27	Up
Intrinsically Disordered Protein, class C 3	idpc-3	2	N	330	-7.01	2.07E-10	N
Dumpy 13 – collagen, downregulated in long-lived, thermally tolerant *npr-8* mutants (Palani et al., 2023)	dpy-13	2	Y	308	-7.94	2.59E-13	Up
A CPG4 domain-containing protein	E01G4.6	2	N	282	-11.66	1.34E-28	N
Roller 1 – collagen, downregulated in long-lived, thermally tolerant *npr-8* mutants (Palani et al., 2023)	rol-1	2	Y	275	-7.79	7.48E-13	Up
A prion-like-(Q/N-rich)-domain-bearing protein	K02E11.10	2	N	262	-9.10	1.99E-17	N
ZK1025.2	ZK1025.2	2	N	241	-6.31	1.91E-08	Up
ZK1025.8	ZK1025.8	2	Y	241	-6.31	1.91E-08	Up
Activated in Blocked Unfolded protein response 1	abu-1	2	N	231	-6.65	2.28E-09	Up
Blistered cuticle 6 – collagen, downregulated in long-lived, thermally tolerant *npr-8* mutants (Palani et al., 2023)	bli-6	2	Y	218	-13.80	1.09E-39	N
Collagen 77 – downregulated in long-lived, thermally tolerant *npr-8* mutants (Palani et al., 2023)	col-77	2	N	208	-11.95	5.09E-30	Up
Collagen 175	col-175	2	N	205	-8.61	1.14E-15	Up
Lipase related 7	lips-7	2	N	203	-6.02	1.03E-07	Up

Table S6. Enriched GO terms among the 199 downregulated DEGs correlated to microbiota colonization. Most enriched annotations indicate genes encoding proteins involved with cuticle and collagen development. Column "p-value" is a multiple-testing adjusted FDR. An additional two InterPro domains and 16 WPO terms are not listed in this table, but similarly relate to collagen biology (Supplemental Data S2).

Annotation type	Term name	Fold-enriched	p-value
GO Biological Process	Cuticle development involved in collagen and cuticulin-based cuticle molting cycle	28	2.82E-7
GO Biological Process	Collagen and cuticulin-based cuticle development	21	5.21E-08
GO Biological Process	Molting cycle, collagen and cuticulin-based cuticle	9	6.49E-5
GO Biological Process	Endoplasmic reticulum unfolded protein response	7	8.96E-5
GO Biological Process	Cellular response to organic substance	4	1.77E-2
GO Biological Process	Anatomical structure development	3	3.66E-12
GO Biological Process	Developmental process	3	4.41E-11
GO Cellular Component	Collagen trimer	32	2.29E-18
GO Cellular Component	Collagen and cuticulin-based cuticle extracellular matrix	29	7.35E-5
GO Cellular Component	External encapsulating structure	10	2.90E-5
GO Cellular Component	Extracellular matrix	9	8.40E-5
GO Molecular Function	Structural constituent of collagen and cuticulin-based cuticle	38	3.73E-5
GO Molecular Function	Structural constituent of cuticle	38	1.33E-79
GO Molecular Function	Structural molecule activity	15	7.66E-58
GO Molecular Function	Extracellular matrix structural constituent	13	3.23E-2

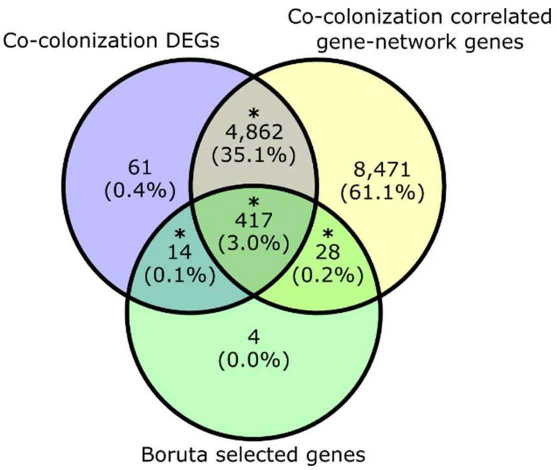

Figure S1. Three analytical methods highlight expression of many of the same host genes associated with co-colonization. Across treatment groups, with emphasis on co-colonization, we detected several thousand genes of interest as DEGs, in co-expression networks correlated to co-colonization, or selected in Boruta random forest analyses. In some cases, a DEG or gene network that is associated with co-colonization may also be additionally correlated to other treatments. We chose to further investigate genes that satisfied at least two of our tests (*) and all genes confirmed by Boruta, as it was the most stringent analysis. Percents given in the diagram are the percent of the total number of genes represented in the figure.

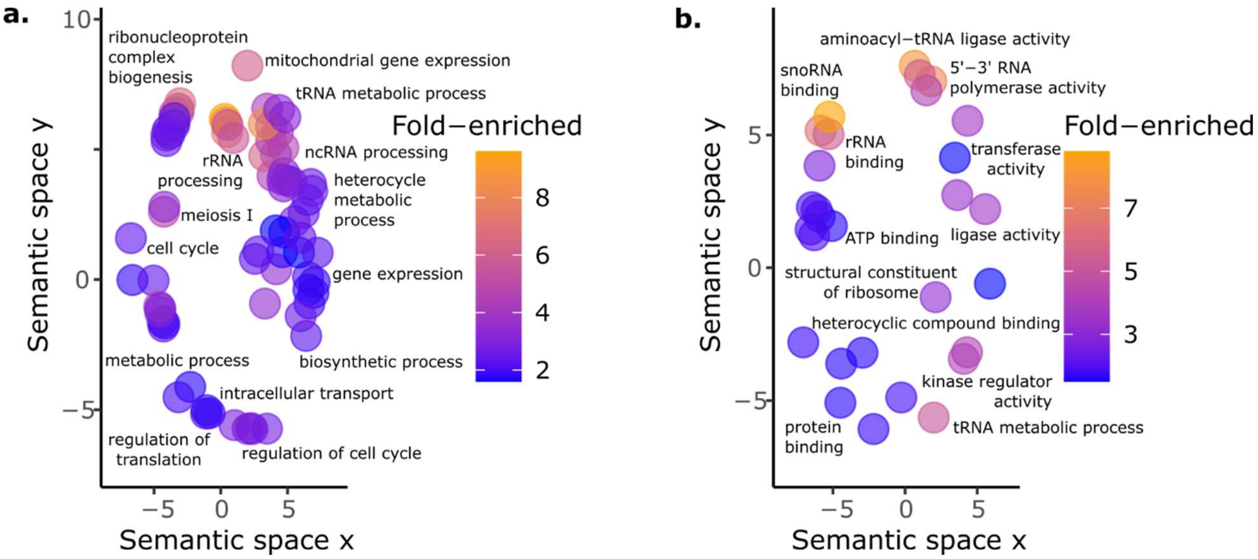

Figure S2. GO term enrichments in WGCNA network 17, which is positively correlated to co-colonization and negatively correlated to pathogen treatment alone. Although numerous these overrepresented annotations largely reflected related processes elements of cell cycle, metabolism, transcription, and translation. Points are labeled for legibility and to show important or representative annotations. GO terms (a.) “peptide biosynthetic process” and “tRNA aminoacylation for protein translation” and (b “DNA-directed 5'-3' RNA polymerase activity” are not depicted as they were above the 0.9 similarity threshold with other terms, Color indicates the fold-increase of overrepresentation

within the test set of genes relative to background. Results are plotted in unitless semantic space, which reflects relatedness within the GO hierarchy, such that more similar terms are clustered together.

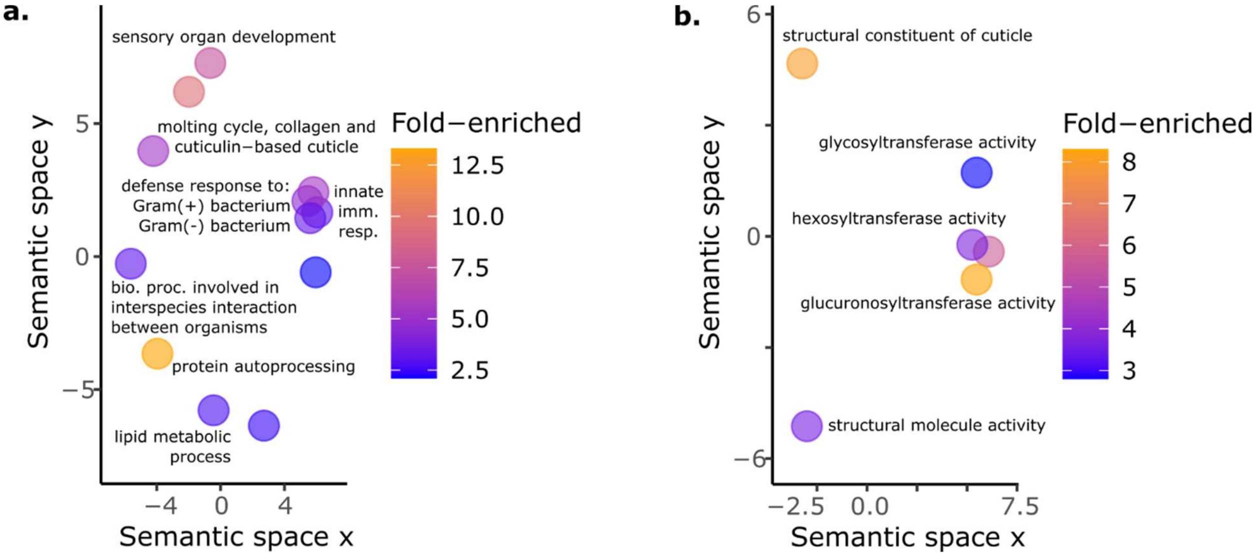

Figure S3. GO terms enriched among genes selected by Boruta, the most conservative experiment-wide analysis. Enriched (a.) Biological Process and (b.) Molecular Function terms reveal changes related to immune function, collagen and development, lipid metabolism, and glycan modification distinguish the host transcriptional response during *S. aureus* infection, microbiota colonization, and/or co-colonization.

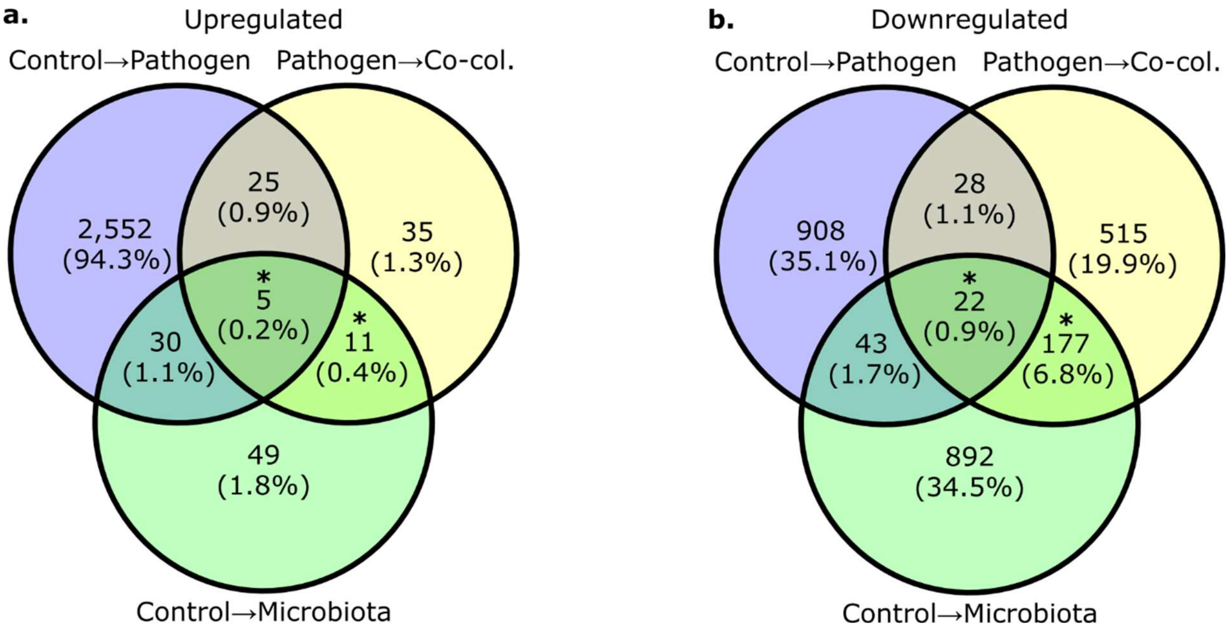

Figure S4. Genes that are uniquely induced by the microbiota even before pathogen invasion may include important players in understanding changes in host condition that underly the difference in mortality between co-colonization and pathogen treatments. (a). Sixteen genes (*) are upregulated by the presence of a

microbiota-only relative to controls and during co-colonization relative to pathogen-only. (b.) 199 genes (*) are downregulated by the presence of a microbiota-only relative to controls and during co-colonization relative to pathogen-only. Co-colonization is abbreviated as “Co-col.”

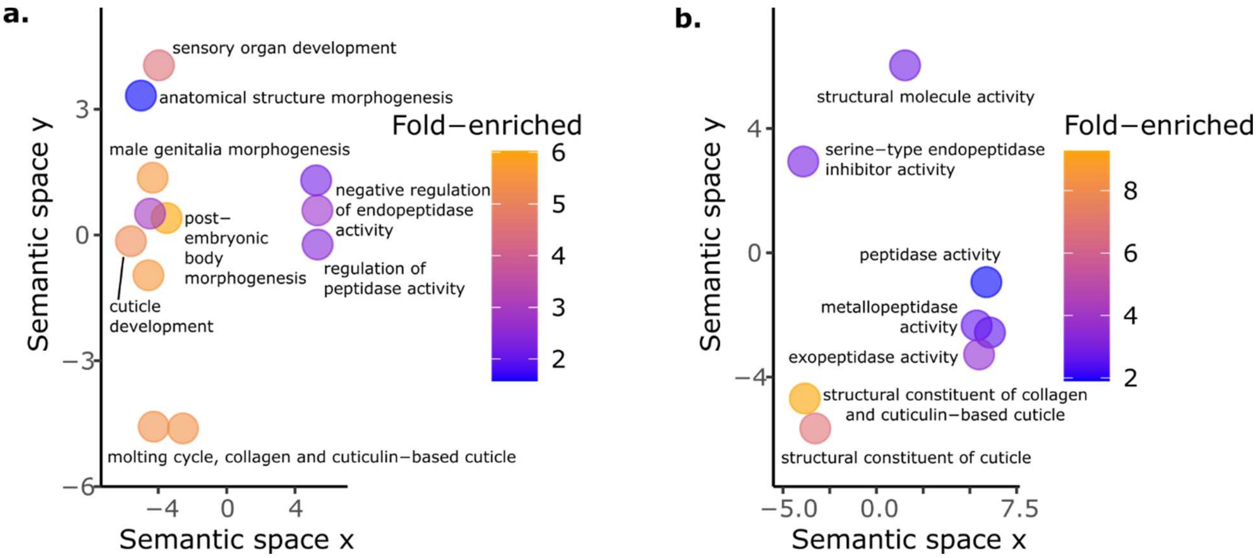

Figure S5. GO term enrichments in WGCNA network 2, which is negatively correlated to co-colonization and positively correlated to pathogen treatment alone. Predominantly, network 2 enrichments relate to host cuticle and collagen development and regulation of peptidase activity. Points are labeled for legibility and to show important or representative annotations. GO terms (a.) “nematode male tail mating organ morphogenesis” and (b.) “DNA-directed 5'-3' RNA polymerase,” “enzyme inhibitor activity,” “endopeptidase inhibitor activity,” and “peptidase inhibitor activity” are not depicted as they were above the 0.9 similarity threshold with other terms, Color indicates the fold-increase of overrepresentation within the test set of genes relative to background. Results are plotted in unitless semantic space, which reflects relatedness within the GO hierarchy, such that more similar terms are clustered together.

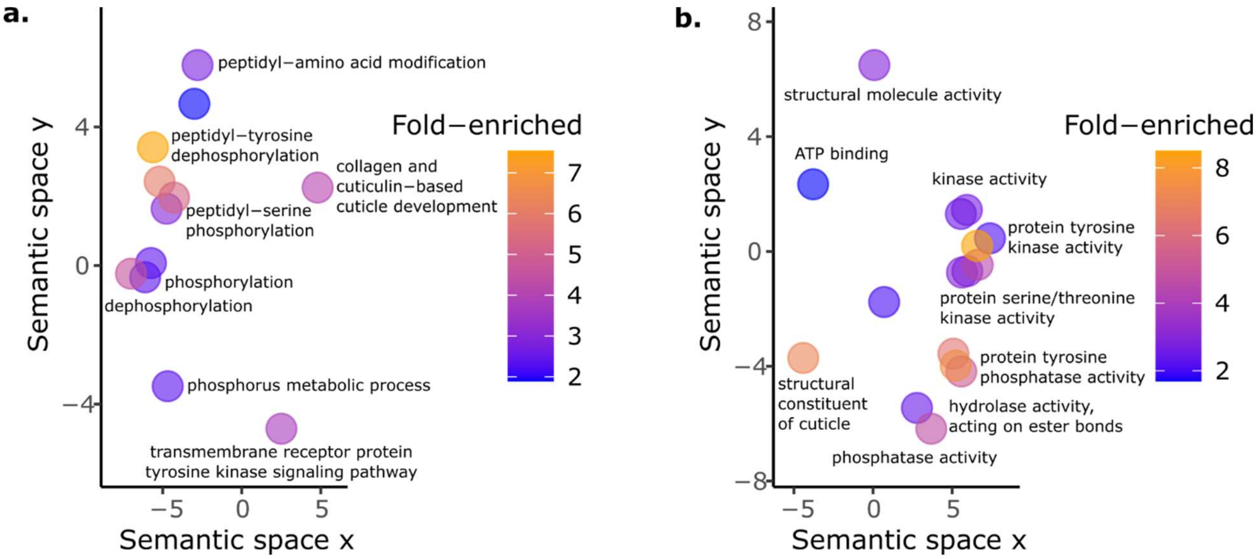

Figure S6. GO term enrichments from DEGs downregulated from controls to microbiota colonization. Most enriched annotations indicate genes encoding proteins involved with protein modification and some related to cuticle and collagen proteins in both (a.) Biological Process and (b.) Molecular Function. Points are labeled for legibility and to show important or representative annotations.

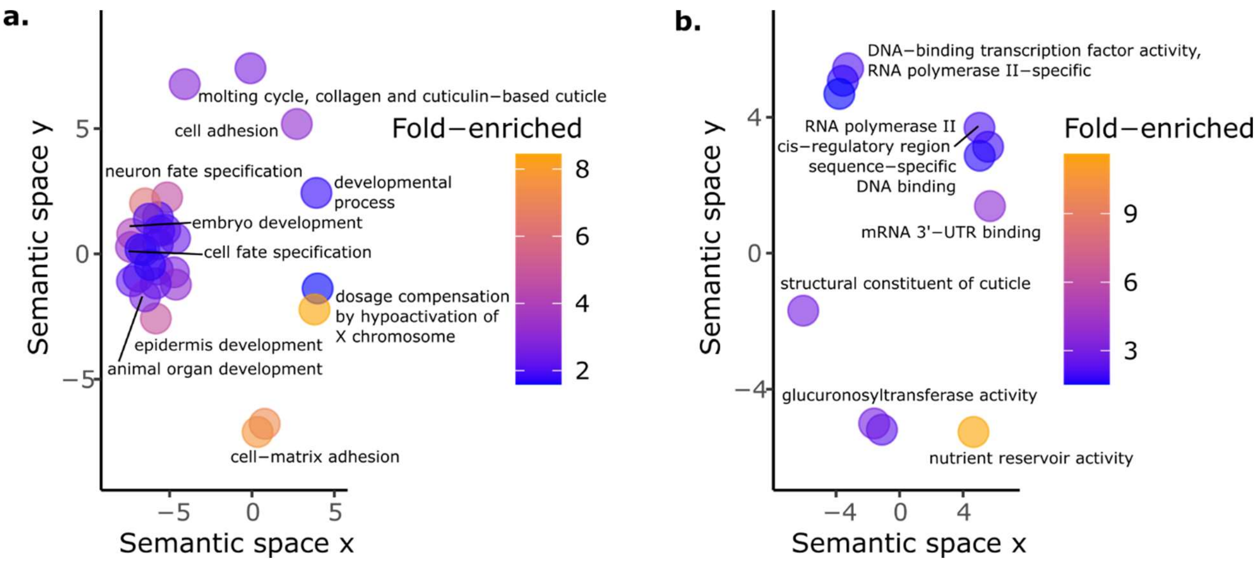

Figure S7. GO term enrichments from DEGs downregulated from microbiota colonization to co-colonization. Early development and reproduction is the major shift from microbiota to co-colonization treatments, but a single term per GO (a.) Biological Process and (b.) Molecular Function implicates changes of collagen/cuticle biology in the host.

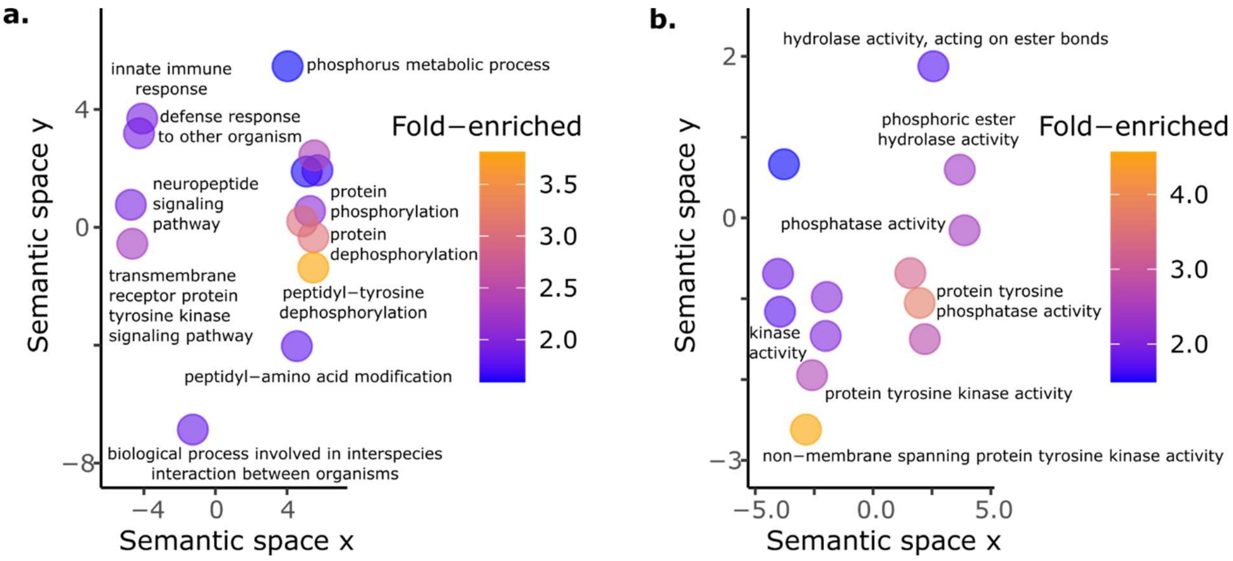

Figure S8. GO term enrichments from DEGs upregulated from microbiota colonization to co-colonization. Protein modification and processing is the major shift from microbiota to co-colonization treatments, but immune response genes are also enriched.

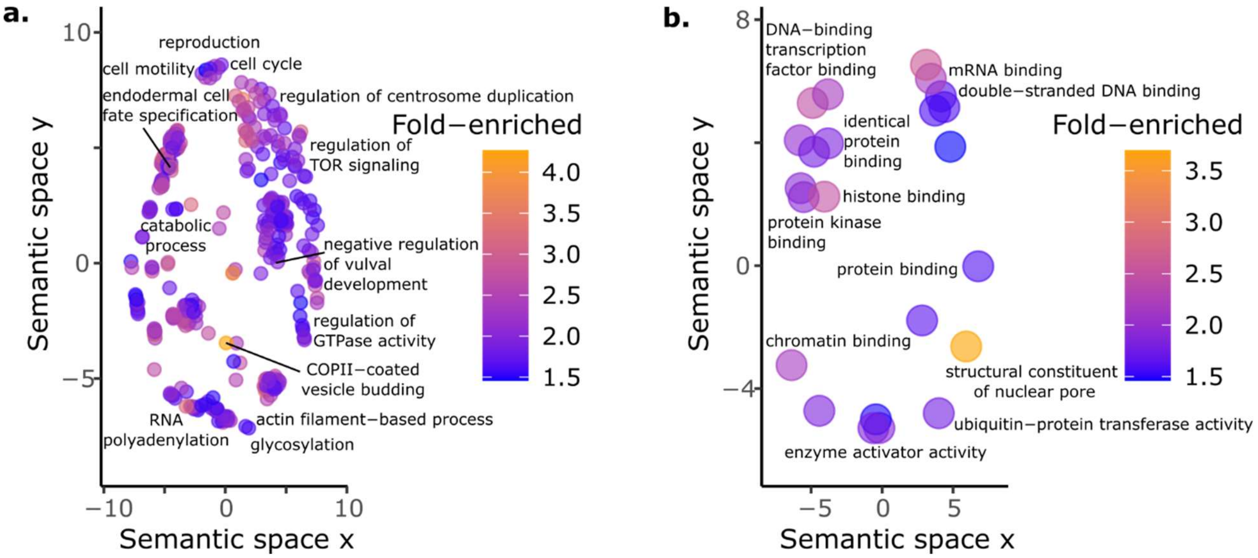

Figure S9. GO term enrichments in WGCNA network 8, which is positively correlated to microbiota treatment and negatively correlated to co-colonization pathogen-only infections. Predominantly, network 8 enrichments relate to cell cycle, development, and reproduction processes seemingly mediated by changes in transcription and protein modification.

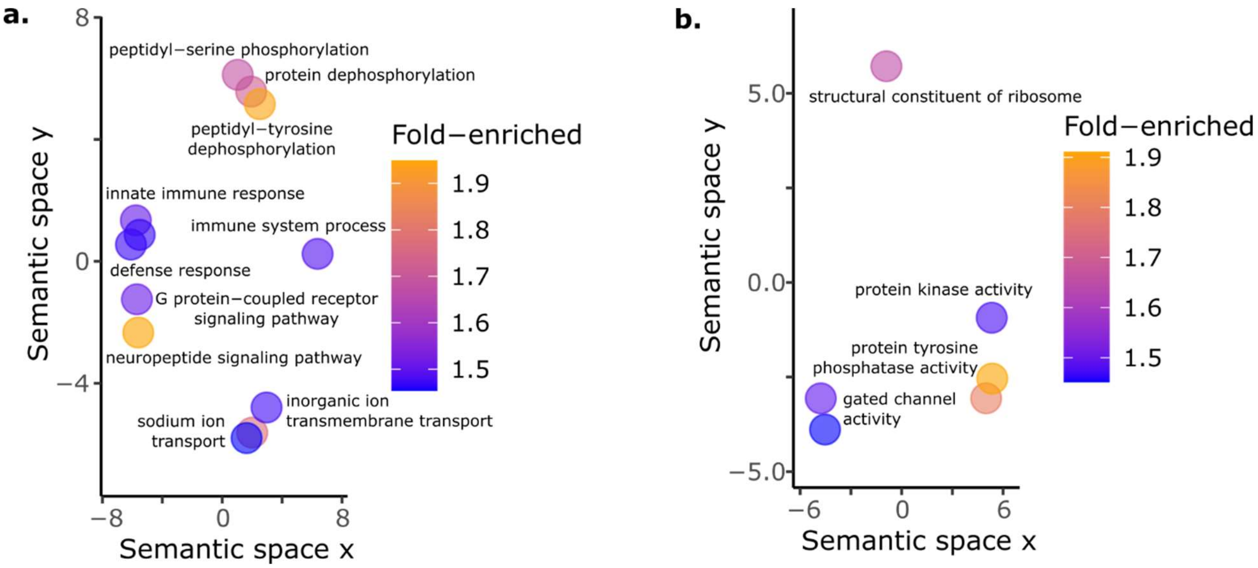

Figure S10. GO term enrichments in WGCNA network 14, which is negatively correlated to microbiota treatment and positively correlated to co-colonization and pathogen-only infections. Predominantly, network 14 enrichments relate protein modification, cell signaling, and immune defense.

Supplemental Document S1. Experiments with evolved bacteria.

Stevens et al., 2024 found that both host-adapted pathogens or microbiota communities facilitated higher levels of infection virulence than their lab-adapted counterparts. As such, we investigated if host transcription could reveal how these differences in virulence arise. To produce these evolved lines, both the microbiota and pathogen were independently serially passaged 15 times through *C. elegans* hosts either *in vivo* to produce host-adapted or *in vitro* to produce lab-adapted lines (Stevens et al., 2024) (Supplemental Document S1: Figure 1). Stevens et al., 2024 passaged host-adapted microbes from colonized worms that were surface sterilized and then crushed to extract gut microbes. Using selective plates (xylose lysine deoxycholate for the microbiota, mannitol salt agar for the pathogen), 100 colonies were picked to inoculate liquid medium, and the liquid culture was used for the next passage through *C. elegans* (Stevens et al., 2024). Lab-adapted lines were produced similarly, but were cultured through liquid media rather than host worms. Lab-adapted lines served as a control for selection during culturing and colony picking. We assayed hosts co-colonized by evolved microbiota (host-adapted or lab-adapted lineages) and ancestral *S. aureus*. For evolved pathogen (host-adapted or lab-adapted) treatments, we only assayed these worms without microbiota co-colonization (Supplemental Document S1: Figure 1). The methods of these assays follow the same procedures as the main experiments with the ancestral bacteria, only substituting evolved bacteria where specified.

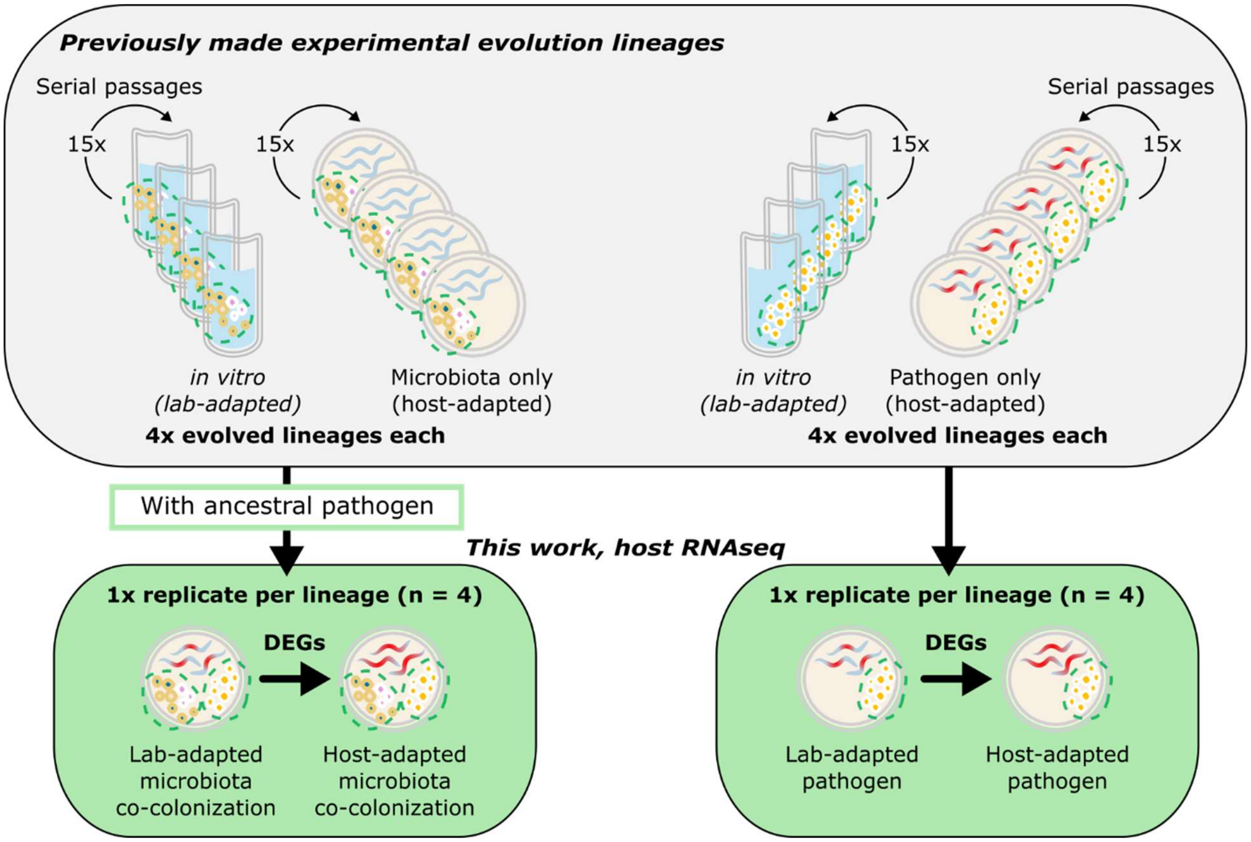

Supplemental Document S1: Figure 1. Evolution experiments design. Each of the four lineages per treatment was started from a common stock of ancestral microbiota or pathogen, then independently passaged for 15 passages. Evolved microbiota co-colonization assay replicates used the same ancestral pathogen for the RNAseq experiments. Lab-adapted treatments were used as control treatments relative to their respective host-adapted treatments. A four-replicate set of *E. coli*-only controls used to estimate RNA extraction batch effects is not depicted.

Using the final evolved populations after 15 serial passages, we performed infection assays and RNA extractions for all evolved treatment samples as a separate batch from ancestral samples. Evolved microbe infection assays and host gene expression analysis followed the methods for the ancestral treatments, with a few key distinctions. A second set of four *E. coli* control treatment extractions were included to estimate batch effects due to husbandry or RNA extraction. All samples were included in the same sequencing run and batch effects between control worms in the first and second extractions appear small (Supplemental Document S1: Figure 2). We were not able to confidently apply WGCNA or Boruta analyses to data from evolved treatments, possibly due to a combination of small effect size and heterogeneity within and between the four evolutionary lineages selected per treatment (Supplemental Document S1: Figure 2). Therefore, we limited comparisons with evolved lines to a cursory search for DEGs between evolved treatments.

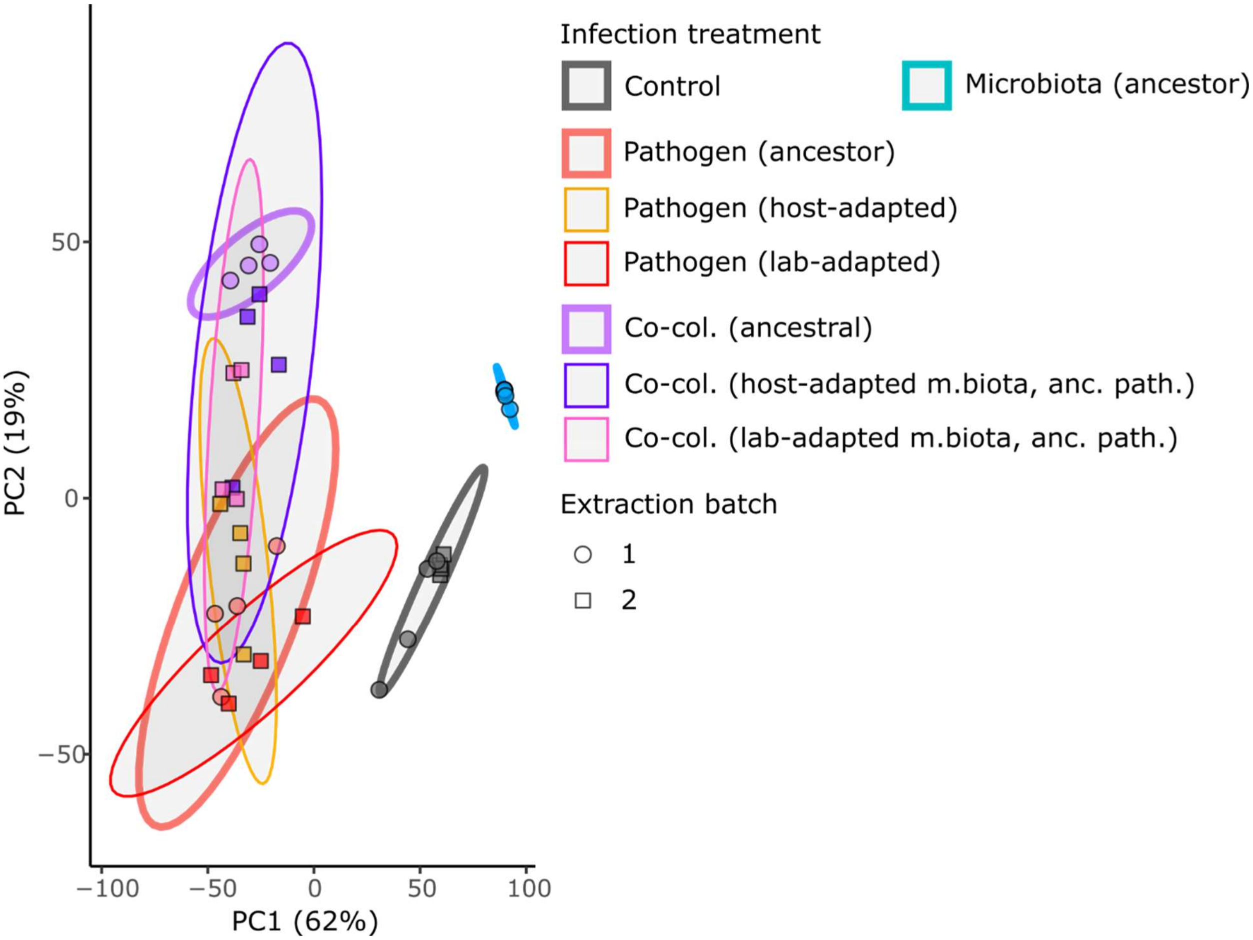

Supplemental Document S1: Figure 2. Control samples show minimal batch variation and treatments with evolved bacteria are difficult to clearly separate. All ancestral sample RNA was collected in batch 1 and treatments with evolved bacteria in batch 2, with controls worms in both batches. Principal components other than 1 and 2 did not readily reveal biologically informative clustering patterns. Co-colonization is abbreviated at "Co-col.", microbiota as "m.biota", and ancestral pathogen as "anc. path.".

Our findings suggest that *C. elegans* hosts may respond similarly to the presence or absence of *S. aureus* and the microbiota, regardless of recent evolutionary history. For pathogen infection alone, Stevens et al., 2024 found higher host mortality with exposure to experimentally evolved host-adapted *S. aureus* pathogens than to experimentally evolved lab-adapted pathogens (albeit, with seemingly low overall virulence in either treatment). However, we did not detect any DEGs distinguishing the host response between these two types of evolved pathogens. Similarly, the host-adapted microbiota was more costly to hosts than lab-adapted microbiota during co-colonization (Stevens et al., 2024). However, we again did not find a clear difference in host transcriptional responses to host-adapted or lab-adapted microbiota co-colonization treatments. These results suggest the evolved microbes can directly shift infection severity without eliciting a substantial, conserved change in host response. Notably, Stevens et

al., 2024 showed that the host-adapted pathogen increases its expression of a core virulence gene (without changing pathogen load) and that the evolved microbiota community structure shifts. These findings offer some insight into how microbes can affect disease outcomes in host-independent manners. However, as there will be heterogeneity within and between evolved lineages of bacteria, these comparisons may be especially impacted by variation among experimental replicates. To what extent these null findings indicate that hosts respond very similarly to evolved variants or that differences require increased statistical power to tease out, remains unclear.

Supplemental Document S2. Culturing and infection assay methods.

Microbe and nematode culturing

Following the methods of Stevens et al., 2024, we grew working cultures of all microbes and *C. elegans* prior to creating experimental treatment plates. We grew cultures of *E. coli* and the seven microbiota species individually in 10 mL LB broth, each inoculated with a single colony and incubated at 25 °C with shaking at 150 rpm. For *S. aureus*, single colonies were grown in 10 mL Todd Hewitt broth at 30 °C with shaking at 150 rpm. The single colonies used for inoculation from the microbiota and *E. coli* were taken from LB agar plates, and the *S. aureus* colonies from tryptic soy agar plates.

To isolate and sterilize eggs, we suspended gravid *C. elegans* worms in 6 mL M9 buffer containing 0.1% Triton-X (M9-Tx) and incubated them with 1 mL bleach lysis solution (1:1 mix of 5 M sodium hydroxide and sodium hypochlorite) for 10 min at room temperature. To collect eggs, we centrifuged (2 mins, 400 x g) and washed pellets twice with M9-Tx. Eggs were then resuspended in M9 and incubated at 20 °C overnight (shaking 150 rpm). Newly hatched L1 larvae arrest in the absence of food, thereby producing an age-synchronized population of *C. elegans*.

Infection of nematode hosts

We exposed age-synchronized worms to control (*E. coli*), microbiota only, pathogen only, or microbiota-pathogen co-colonization treatments. All *C. elegans* were first grown on a “food” plate prior to being transferred to an “assay” plate. Microbiota and co-colonization treatment food plates consisted of 9 cm nematode growth medium (NGM) agar plates with a microbiota lawn. To produce microbiota food plates, we standardized the working cultures (see above) of each species to optical density (600 nm) of the least turbid culture. Then, we inoculated NGM plates with 600 µL of an equal-volume pool of all seven microbiota species. Control and pathogen-only food plates consisted of 600 µL *E. coli*, at the same optical density as the standardized microbiota cultures. All food plates were air dried and then we added approximately 1,000 L1 *C. elegans* per plate. The worms on food plates were incubated at 20 °C for 48 h.

While the L1 *C. elegans* incubated on food plates, we grew assay plates. Using the same working cultures as used to inoculate food plates, we spread 100 µl of either the microbiota pool, *E. coli*, or *S.*

aureus onto NGM agar plates. Assay plates were incubated for 24 h, at 20 °C for the microbiota, or 30 °C for *E. coli* and *S. aureus*. Assay plates were then stored at 4 °C until worms were ready to be transferred from food plates.

After the 48 h incubation on food plates, we harvested worms off plates with 850 µl M9-Tx and washed them twice with 250 µl M9-Tx (2 mins, 290 x g centrifugation). Worms were suspended in a final volume of 100 µl M9-Tx and spotted onto assay plates. Control worms were transferred from *E. coli* food plates to *E. coli* assay plates. Microbiota-only worms were transferred from microbiota food plates to microbiota assay plates. Pathogen-only worms were transferred from *E. coli* food plates to *S. aureus* assay plates. Co-colonization worms were transferred from microbiota food plates to *S. aureus* assay plates. All assay plates were incubated at 25 °C for 12 h prior to sampling for RNAseq.

References cited

Barsyte, D., Lovejoy, D. A., & Lithgow, G. J. (2001). Longevity and heavy metal resistance in daf-2 and age-1 long-lived mutants of Caenorhabditis elegans. *The FASEB Journal*, *15*(3), 627–634. https://doi.org/10.1096/FJ.99-0966COM

Brunquell, J., Morris, S., Lu, Y., Cheng, F., & Westerheide, S. D. (2016). The genome-wide role of HSF-1 in the regulation of gene expression in Caenorhabditis elegans. *BMC Genomics*, *17*(1). https://doi.org/10.1186/S12864-016-2837-5

Dierking, K., Yang, W., & Schulenburg, H. (2016). Antimicrobial effectors in the nematode Caenorhabditis elegans: an outgroup to the Arthropoda. *Philosophical Transactions of the Royal Society B: Biological Sciences*, *371*(1695). https://doi.org/10.1098/RSTB.2015.0299

Fanelli, M. J., Welsh, C. M., Lui, D. S., Smulan, L. J., & Walker, A. K. (2023). Immunity-linked genes are stimulated by a membrane stress pathway linked to Golgi function and the ARF-1 GTPase. *Science Advances*, *9*(49), eadi5545. https://doi.org/10.1126/SCIADV.ADI5545

Ford, S. A., Drew, G. C., & King, K. C. (2022). Immune-mediated competition benefits protective microbes over pathogens in a novel host species. *Heredity*, *129*(6), 327. https://doi.org/10.1038/S41437-022-00569-3

Garrigues, J. M., Tsu, B. V., Daugherty, M. D., & Pasquinelli, A. E. (2019). Diversification of the Caenorhabditis heat shock response by helitron transposable elements. *ELife*, *8*. https://doi.org/10.7554/ELIFE.51139

Kim, D. H., Liberati, N. T., Mizuno, T., Inoue, H., Hisamoto, N., Matsumoto, K., & Ausubel, F. M. (2004). Integration of Caenorhabditis elegans MAPK pathways mediating immunity and stress resistance by MEK-1 MAPK kinase and VHP-1 MAPK phosphatase. *Proceedings of the National Academy of Sciences of the United States of America*, *101*(30), 10990–10994. https://doi.org/10.1073/PNAS.0403546101

Madhu, B., Lakdawala, M. F., & Gumienny, T. L. (2023). The DBL-1/TGF-β signaling pathway tailors behavioral and molecular host responses to a variety of bacteria in Caenorhabditis elegans. *ELife*, *12*. https://doi.org/10.7554/ELIFE.75831

Mertensкötter, A., Keshet, A., Gerke, P., & Paul, R. J. (2013). The p38 MAPK PMK-1 shows heat-induced nuclear translocation, supports chaperone expression, and affects the heat tolerance of Caenorhabditis elegans. *Cell Stress & Chaperones*, *18*(3), 293. https://doi.org/10.1007/S12192-012-0382-Y

Murphy, C. T., McCarroll, S. A., Bargmann, C. I., Fraser, A., Kamath, R. S., Ahringer, J., Li, H., & Kenyon, C. (2003). Genes that act downstream of DAF-16 to influence the lifespan of Caenorhabditis elegans. *Nature 2003 424:6946*, *424*(6946), 277–283. https://doi.org/10.1038/nature01789

Palani, S. N., Sellegounder, D., Wibisono, P., & Liu, Y. (2023). The longevity response to warm temperature is neurally controlled via the regulation of collagen genes. *Aging Cell*, *22*(5), e13815. https://doi.org/10.1111/ACEL.13815

Peterson, N. D., Cheesman, H. K., Liu, P., Anderson, S. M., Foster, K. J., Chhaya, R., Perrat, P., Thekkiniath, J., Yang, Q., Haynes, C. M., & Pukkila-Worley, R. (2019). The nuclear hormone receptor NHR-86 controls anti-pathogen responses in C. elegans. *PLoS Genetics*, *15*(1). https://doi.org/10.1371/JOURNAL.PGEN.1007935

Pukkila-Worley, R., Feinbaum, R. L., McEwan, D. L., Conery, A. L., & Ausubel, F. M. (2014). The Evolutionarily Conserved Mediator Subunit MDT-15/MED15 Links Protective Innate Immune Responses and Xenobiotic Detoxification. *PLoS Pathogens*, *10*(5). https://doi.org/10.1371/JOURNAL.PPAT.1004143

Rajan, M., Anderson, C. P., Rindler, P. M., Romney, S. J., Dos Santos, M. C. F., Gertz, J., & Leibold, E. A. (2019). NHR-14 loss of function couples intestinal iron uptake with innate immunity in C. elegans through PQM-1 signaling. *ELife*, *8*. https://doi.org/10.7554/ELIFE.44674

Roberts, A. F., Gumienny, T. L., Gleason, R. J., Wang, H., & Padgett, R. W. (2010). Regulation of genes affecting body size and innate immunity by the DBL-1/BMP-like pathway in Caenorhabditis elegans. *BMC Developmental Biology*, *10*(1), 1–10. https://doi.org/10.1186/1471-213X-10-61/FIGURES/1

Romney, S. J., Thacker, C., & Leibold, E. A. (2008). An Iron Enhancer Element in the FTN-1 gene directs iron-dependent expression in Caenorhabditis elegans intestine. *Journal of Biological Chemistry*, *283*(2), 716–725. https://doi.org/10.1074/jbc.M707043200

Shapira, M., Hamlin, B. J., Rong, J., Chen, K., Ronen, M., & Tan, M. W. (2006). A conserved role for a GATA transcription factor in regulating epithelial innate immune responses. *Proceedings of the National Academy of Sciences of the United States of America*, *103*(38), 14086–14091. https://doi.org/10.1073/PNAS.0603424103

Shim, J., Im, S. H., & Lee, J. (2003). Tissue-specific expression, heat inducibility, and biological roles of two hsp16 genes in Caenorhabditis elegans. *FEBS Letters*, *537*(1–3), 139–145. https://doi.org/10.1016/S0014-5793(03)00111-X

Singh, V., & Aballay, A. (2006). Heat-shock transcription factor (HSF)-1 pathway required for Caenorhabditis elegans immunity. *Proceedings of the National Academy of Sciences of the United States of America*, *103*(35), 13092. https://doi.org/10.1073/PNAS.0604050103

Stevens, E. J., Li, J. D., Hector, T. E., Drew, G. C., Greenrod, S. T. E., Paterson, S., & King, K. C. (2024). Within-host competition sparks pathogen molecular evolution and perpetual microbiota dysbiosis. *BioRxiv Preprint*. https://doi.org/doi.org/10.1101/2024.09.03.610829

Tvermoes, B. E., Boyd, W. A., & Freedman, J. H. (2010). Molecular characterization of numr-1 and numr-2: genes that increase both resistance to metal-induced stress and lifespan in Caenorhabditis elegans. *Journal of Cell Science*, *123*(12), 2124. https://doi.org/10.1242/JCS.065433

Wong, D., Bazopoulou, D., Pujol, N., Tavernarakis, N., & Ewbank, J. J. (2007). Genome-wide investigation reveals pathogen-specific and shared signatures in the response of Caenorhabditis elegans to infection. *Genome Biology*, *8*(9), 1–18. https://doi.org/10.1186/GB-2007-8-9-R194
